# NMDA Receptor Mediated Mechanisms in the Post-Stroke Brain: From Physiology to Pathology

**DOI:** 10.3390/biom16060770

**Published:** 2026-05-23

**Authors:** Han Gong, Xiang-Zheng Wang, Dan Liu, Wei-Jin Liu, Xiao-Xia Du, Jia-Sheng Rao

**Affiliations:** 1 Beijing Key Laboratory for Biomaterials and Neural Regeneration, National Medical Innovation Platform for Industry-Education Integration in Advanced Medical Devices (Interdiscipline of Medicine and Engineering), School of Biological Science and Medical Engineering, Beihang University, Beijing 100191, China; 2 China Rehabilitation Research Center, Beijing 100086, China; 3 China Rehabilitation Science Institute, Beijing 100086, China; 4 School of Rehabilitation Sciences and Engineering, University of Health and Rehabilitation Sciences, Qingdao 266113, China; 5 Beijing International Cooperation Bases for Science and Technology on Biomaterials and Neural Regeneration, Beijing Advanced Innovation Center for Biomedical Engineering, Beihang University, Beijing 100191, China; 6 School of Rehabilitation Medicine, Wenzhou Medical University, Wenzhou 325000, China; 7 School of Rehabilitation, Capital Medical University, Beijing 100086, China

**Keywords:** NMDA receptor, ischemic stroke, excitotoxicity, pathophysiology, neuroplasticity, neuroprotection, excitation-inhibition balance

## Abstract

N-methyl-D-aspartate receptors (NMDARs) play a context-dependent role in ischemic stroke (IS), contributing to acute excitotoxic injury while also supporting subsequent neuroplasticity. This functional divergence has constrained the therapeutic efficacy of non-selective NMDAR antagonists. During the acute phase, neuronal injury is associated with the redistribution of NMDARs toward extrasynaptic sites and the activation of aberrant non-ionotropic signaling pathways. As the disease progresses, NMDAR-dependent signaling becomes increasingly involved in activity-dependent plasticity, including motor engram consolidation, dendritic remodeling, and large-scale network reorganization. Post-stroke cognitive impairment and depression are increasingly recognized as potential consequences of sustained NMDAR dysregulation, involving interactions with immune signaling and metabolic processes. These observations support a shift toward activity-dependent modulation of NMDAR function, in which neurotoxic signaling is selectively dissociated from physiological receptor activity. Emerging strategies aimed at subunit-specific modulation and disruption of pathological receptor complexes provide a basis for more targeted intervention. Preservation of physiological excitation–inhibition balance may therefore represent a key requirement for optimizing functional recovery after stroke.

## 1. Introduction

Ischemic stroke (IS) is a leading cause of long-term disability globally, often leading to persistent motor, cognitive, and emotional dysfunction [1,2,3]. Beyond the acute focal infarction induced by cerebral ischemia, stroke also disrupts the neural circuits that support coordinated brain function and recovery. Among the molecular mechanisms governing these processes, N-methyl-D-aspartate receptors (NMDARs) play a central role in excitatory synaptic transmission and activity-dependent neuroplasticity [4,5]. As glutamate-gated ion channels with high calcium permeability, NMDARs play a pivotal role in regulating synaptic strength, neuronal survival, and circuit remodeling [6].

Following ischemic stroke, NMDAR signaling exhibits a complex biphasic pattern. During the acute phase, excessive NMDAR activation contributes to excitotoxic calcium influx, leading to neuronal injury and cell death [7]. In contrast, during the subacute and chronic phases, physiological NMDAR signaling becomes essential for synaptic remodeling, circuit reorganization, and the consolidation of motor engrams underlying functional recovery [8]. These temporally distinct roles highlight the importance of tightly regulated receptor activity in the evolving pathophysiology of stroke.

Given this pivotal role, modulation of NMDAR activity has long been investigated as a potential therapeutic strategy for ischemic stroke. Although conventional non-selective NMDAR antagonists can effectively reduce acute excitotoxic injury and infarct volume, their capacity to improve long-term functional recovery remains severely limited [9].

Therefore, this review aims to present a comprehensive narrative synthesis of current evidence concerning NMDAR function following ischemic stroke. To establish a rigorous scope, a targeted literature search was conducted in PubMed for English-language articles published through March 2026. References were selected based on their direct relevance to NMDAR-mediated neuroplasticity, molecular pathophysiology, and clinical translation in stroke rehabilitation. Particular attention is devoted to how receptor properties, downstream signaling mechanisms, and stage-specific alterations influence post-stroke neuronal plasticity, circuit reorganization, and functional recovery. By integrating findings from molecular, cellular, and systems-level studies, this review aims to clarify the multifaceted role of NMDARs in post-stroke dysfunction and functional recovery.

## 2. Structural Foundations and Gating Kinetics of NMDARs

Ionotropic glutamate receptors (iGluRs) are classified into four distinct classes based on their pharmacological properties and structural homology. These include α-amino-3-hydroxy-5-methyl-4-isoxazolepropionic acid (AMPA) receptors (GluA1–GluA4), kainate (KA) receptors (GluK1–GluK5), NMDA receptors (GluN1, GluN2A–D, and GluN3A–B), and δ-receptors (GluD1 and GluD2) [10,11]. Among these, AMPA, kainate, and NMDA receptors represent canonical ligand-gated cation channels. Excitatory synaptic transmission is primarily mediated by these receptors, which are also critically involved in regulating neuronal excitability and synaptic plasticity [12].

The NMDA receptor is a heterotetrameric assembly typically composed of two GluN1 and two GluN2 subunits [13]. Furthermore, the kinetic properties, pharmacological sensitivity, and signal transduction patterns of NMDARs are critically determined by the specific type of the incorporated GluN2 subunit [6]. Each subunit is composed of four distinct domains: an amino-terminal domain (ATD/NTD), a ligand-binding domain (LBD), a transmembrane domain (TMD), and a carboxyl-terminal domain (CTD). The ATD and LBD are extracellular, whereas the ion channel pore is formed by the TMD [14,15]. Critical intracellular interactions with signaling and scaffolding proteins are mediated by the CTD [16].

### 2.1. Cross-Domain Conformational Coupling and Gating Intermediates

The gating process of NMDARs relies on cross-domain conformational coupling. Following the binding of glutamate and glycine to the LBD, the mechanical tension generated by LBD closure is transmitted to the transmembrane region via LBD–TMD linkers, thereby driving the conformational rearrangement of the TMD. The channel domain displays pseudo-fourfold symmetry in the resting state but rearranges into a twofold symmetric configuration upon channel opening [17,18]. The activation of NMDARs is typically completed within approximately 10 ms, whereas the deactivation kinetics are primarily dictated by the GluN2 subunit [19]. Channel opening does not occur instantaneously but instead proceeds through multiple pre-active intermediate states. This process involves the concerted movement of the Pre-M1 and S2–M4 linkers, which progressively relieve conformational constraints on the pore-lining M3 helix [20]. This helix undergoes asymmetric bending to form a transient open conformation, which is stabilized by reconstituted interactions between the D2–M3 linker and the surrounding structural elements.

Distinct GluN2 subtypes exhibit different allosteric signaling pathways [21]. While the ATD of GluN2A adopts a rigid conformation, that of GluN2B shows conformational flexibility [22]. Allosteric signals in GluN2A are transmitted through the D1–D1 dimer interface, whereas GluN2B-dependent signaling depends on rolling motions between LBD dimers [23]. Furthermore, the kinetic properties, pharmacological sensitivity, and signal transduction patterns of NMDARs are critically determined by the specific type of the incorporated GluN2 subunit [6].

NMDAR gating is governed by a transmembrane two-gate mechanism [24]. The M4 helix of the GluN2 subunit modulates canonical M3 gating, thereby regulating the sustained channel activation of the receptor. In contrast, the dynamics of the GluN1 M2 loop regulate ion permeation and gating kinetics, together establishing a hierarchical and coordinated gating mechanism [21]. The channel open probability of the receptor and its coupling efficiency with downstream signaling complexes are significantly modulated by the phosphorylation state of the GluN2A and GluN2B subunits [14]. In addition, the functional diversity of NMDARs is shaped by post-translational modifications and interactions with associated proteins [25].

### 2.2. The Role of Subunit Switching in Synaptic Maturation

During early brain development, particularly in the embryonic stage, the GluN2B subunit predominates within NMDARs. During postnatal maturation, NMDAR subunit composition undergoes a well-characterized developmental switch from GluN2B dominance to GluN2A dominance [26,27]. This transition does not represent a simple subunit replacement but instead involves a coordinated remodeling of receptor functional properties. Specifically, GluN2B-containing receptors exhibit prolonged channel open times and slower deactivation kinetics, whereas increased incorporation of GluN2A confers faster response kinetics and enhanced temporal precision [28]. Consequently, this developmental shift reshapes NMDAR Ca^2+^ conductance kinetics and downstream intracellular signaling processes. These processes are differentially governed by distinct intracellular domains of GluN2 subunits and are temporally coupled to the maturation of dendritic architecture and synaptic plasticity. This subunit transition facilitates the progression of neural circuits from a highly plastic early state toward a more stable and efficient mode of information processing.

Although GluN2B is also present at synapses, it is predominantly localized to extrasynaptic regions and is closely associated with neuronal apoptosis and excitotoxicity. The functional differences arising from subunit switching cannot be fully explained by classical Ca^2+^-dependent ionotropic signaling alone. Accumulating evidence indicates that NMDAR signaling operates through three interconnected dimensions: ionotropic Ca^2+^-dependent signaling, non-ionotropic conformational signaling, and scaffold-mediated signaling complex coupling [29,30]. In the canonical ionotropic pathway, Ca^2+^ influx through NMDARs binds to CaM and activates CaMKII, which undergoes autophosphorylation at Thr286 to achieve sustained activation. This process enables CaMKII to bind the GluN2B CTD, forming a stable CaMKII–GluN2B complex that prolongs synaptic signaling and supports the induction and maintenance of LTP [31,32]. Beyond ion flux, NMDARs also signal through a non-ionotropic, conformational mechanism. Glutamate binding induces receptor conformational changes that trigger downstream signaling cascades even when ion permeation is blocked by MK-801. This pathway, mediated by the GluN2B CTD, recruits the p38 MAPK–cofilin axis to drive actin cytoskeletal remodeling within dendritic spines, thereby regulating structural plasticity [33,34]. In parallel, the CTD of GluN2 subunits functions as a signaling hub by anchoring NMDARs to multiprotein complexes via scaffold proteins such as PSD-95. Through this interaction, NMDARs are physically coupled to neuronal nNOS, forming the NMDAR/PSD-95/nNOS complex [35]. While this complex contributes to synaptic plasticity under physiological conditions, it can also mediate excitotoxic neuronal injury via NO-dependent oxidative stress under pathological conditions [36]. Notably, these signaling modalities are not mutually exclusive but operate in parallel, with their relative contributions determined by receptor localization and pathological context [37].

### 2.3. Non-Ionotropic Signaling and Dendritic Remodeling

**Figure 1:**
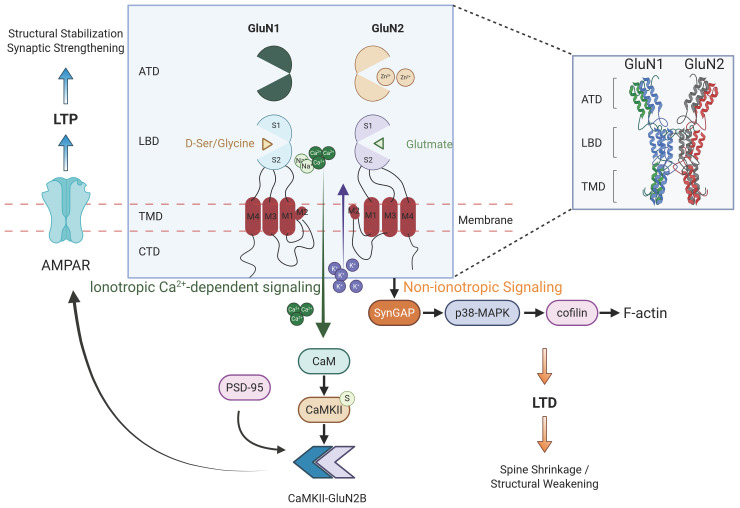
Structural basis of NMDAR and its dual ionotropic and non-ionotropic signaling pathways in bidirectional synaptic plasticity. The NMDAR is a heterotetramer primarily composed of GluN1 and GluN2 subunits. The upper right illustrates its 3D architecture (ATD, LBD, and TMD). Activation of the receptor requires the concurrent binding of glutamate and a co-agonist (D-serine or glycine) to the LBD. (**Left**): Classical ionotropic Ca^2+^-dependent signaling. Receptor opening allows Ca^2+^ influx, which binds to CaM and activates CaMKII. Activated CaMKII forms a complex with GluN2B and PSD-95, promoting the recruitment of AMPARs to the postsynaptic membrane. This cascade induces structural stabilization, synaptic strengthening, and ultimately LTP. (**Right**): Non-ionotropic signaling. Independent of ion flux, ligand binding induces conformational changes that trigger the SynGAP/p38-MAPK/cofilin signaling cascade, leading to F-actin remodeling. This pathway mediates spine shrinkage, structural weakening, and LTD.

Synaptic structural modulation is mediated by NMDARs via non-ionotropic conformational signaling, independent of classical Ca^2+^ permeation. Glutamate binding induces receptor conformational changes that initiate downstream signaling cascades. These cascades are activated even when the ion pore is occluded, and Ca^2+^ influx is abolished by the channel blocker MK-801. This pathway is mediated by the extended CTD of the GluN2B subunit [33]. The binding interaction between the CTD and scaffold proteins, such as SynGAP, is modulated by receptor conformational changes. The p38 MAPK pathway is recruited and activated to regulate the actin-severing protein cofilin. This cascade drives the physical remodeling of filamentous actin (F-actin) in dendritic spines [34]. NMDAR signaling is mediated by a conformation-dependent metabotropic dimension alongside classical ion permeation (Figure 1).

Synaptic signal anchoring is dictated by the structural network of the CTD. The molecular basis for long-term potentiation (LTP) induction is established by CaMKII-NMDAR binding [31]. Following NMDAR-mediated Ca^2+^ influx, Ca^2+^ binds to calmodulin (CaM). The resulting Ca^2+^/CaM complex activates CaMKII and induces autophosphorylation at Thr286 [38]. The GluN2B binding site on CaMKII is exposed by autophosphorylation. A stable CaMKII–GluN2B complex is formed through interaction with the GluN2B CTD. This complex sustains CaMKII activity and prolongs local signaling [39]. The GluN2B CTD engages the core scaffold protein postsynaptic density-95 (PSD-95) within this signaling network. NMDARs are physically coupled to neuronal nitric oxide synthase (nNOS) by PSD-95, a membrane-associated guanylate kinase (MAGUK), to form the tripartite NMDAR/PSD-95/nNOS signaling complex [40]. This complex mediates synaptic plasticity and pathological toxicity. PSD-95 is phosphorylated at Ser73 [32]. AMPA receptor synaptic localization and negative regulation of NMDAR are governed by alterations in PSD-95 stability [41]. Aberrant PSD-95 expression or spatial distribution contributes to the progression of Alzheimer’s disease [42], age-related memory decline [43], and mood disorders [44]. GABAergic inhibitory function in the prefrontal cortex is impaired by PSD-95 deficiency [45].

## 3. Pathophysiology of NMDARs in IS

Physiological synaptic function and neuronal survival are maintained by moderate NMDAR-mediated Ca^2+^ influx. The receptor microenvironment is altered during IS. NMDARs transition from survival-promoting hubs to drivers of excitotoxicity and neuronal apoptosis. This functional reversal is governed by a spatiotemporal pathological evolution, encompassing neurotransmitter spillover, molecular receptor modifications, and network reorganization.

### 3.1. Astrocytic Transporter Failure and Spatial Misallocation

NMDAR-mediated excitotoxicity is initiated by glutamate spillover during the acute ischemic phase [46]. The astrocytic transporters GLT-1 and GLAST utilize the transmembrane Na^+^ gradient to clear extracellular glutamate. The phasic activation of synaptic NMDARs is maintained by this clearance. ATP depletion and the dissipation of transmembrane ion gradients are induced by ischemia, which impairs and functionally reverses these transporters. High glutamate concentrations accumulate in the extrasynaptic space. The tonic activation of extrasynaptic NMDARs (eNMDARs) is driven by this accumulation [47].

The nanoscale organization of membrane NMDARs is altered by the disruption of transmitter homeostasis [48]. The lateral diffusion of NMDARs is restricted by the PSD scaffold protein network. This restriction is attenuated by ischemia-induced depolarization and cytoskeletal rearrangement. NMDARs undergo lateral migration toward extrasynaptic regions. This spatial misallocation increases the proportion of extrasynaptic GluN2B-NMDARs. Downstream signaling is redirected from the cAMP response element-binding protein (CREB) dependent survival pathway to pro-apoptotic cascades [49].

### 3.2. Molecular Mechanisms of Excitotoxicity: PPIs and Phosphorylation

The ischemic microenvironment and subsequent reperfusion injury induce aberrant posttranslational modifications of NMDARs, exacerbating Ca^2+^ overload and neuronal damage [50]. Ischemic stimuli hyperactivate Src-family tyrosine kinases, leading to phosphorylation of Tyr1472 in the GluN2B CTD [51]. This modification exerts dual pathological effects: it increases channel open probability while disrupting AP-2 binding, thereby preventing receptor endocytosis and trapping hyperactive GluN2B-containing NMDARs at the cell surface. Reactive nitrogen species (RNS) generated during vascular recanalization and reperfusion mediate S-nitrosylation of Fyn and NMDARs [52]. These synergistic modifications sustain aberrant GluN2B activation, driving a pathological positive feedback loop of continuous Ca^2+^ influx [53].

Sustained eNMDAR activation and dysregulated CTD modifications drive the rewiring of specific protein–protein interaction (PPI) networks, establishing the molecular basis for cell death. Ischemia promotes the recruitment of DAPK1 to the GluN2B CTD [54], resulting in phosphorylation at Ser1303 and amplification of neurotoxic signaling [55]. Concurrently, pathological eNMDARs assemble with TRPM4 to form functional transmembrane complexes. TRPM4-mediated Na^+^ influx sustains membrane depolarization, relieving the voltage-dependent Mg^2+^ block and thereby exacerbating Ca^2+^ overload [51]. Furthermore, the NMDAR/PSD-95/nNOS ternary complex remains intact under ischemic conditions, promoting excessive nitric oxide (NO) production. NO reacts with superoxide to generate the highly cytotoxic oxidant peroxynitrite, which in turn amplifies nitrative and oxidative stress [40].

NMDAR-mediated ischemic injury extends beyond classical ion overload. In the ischemic penumbra, sublethal glutamate stimulation activates non-ionotropic NMDAR signaling independent of prominent Ca^2+^ influx [56]. Sustained ligand binding induces conformational changes that engage LTD-associated pathways, particularly the p38 MAPK–cofilin axis [57]. Activation of this cascade promotes F-actin depolymerization within dendritic spines, leading to spine shrinkage and synaptic loss. This ultrastructural synaptic degeneration ultimately disrupts the signaling architecture of local cortical circuits [58].

Pathological consequences of IS are not confined to acute focal injury; they evolve into subacute disruption of distributed brain networks [59]. Functional recovery depends on global network reorganization rather than isolated focal compensation [60]. However, eNMDAR overactivation and synaptic atrophy deprive compromised networks of the molecular substrate for physiological remodeling. Neuroimaging and clinical cohorts demonstrate a pathological decline in functional connectivity between motor and non-motor networks during the acute-to-subacute transition [61]. The severity of this disruption accurately predicts long-term motor recovery trajectories [62,63].

Collectively, ischemia-driven NMDAR dysfunction exerts a dual detrimental effect, contributing to acute neuronal injury while simultaneously preventing network reconnection and functional motor recovery.

## 4. NMDAR-Dependent Plasticity and Circuit Reorganization

While acute NMDAR overactivation drives excitotoxicity, tightly regulated signaling is indispensable for structural and system-level network reorganization during recovery [46,64]. As IS transitions into the subacute and chronic phases, reduced tissue toxicity redirects the biological trajectory of vulnerable regions toward tissue repair and functional compensation [65]. During this phase, NMDAR-mediated synaptic remodeling and the functional integration of large-scale brain networks form the essential molecular basis for neuroplasticity, supporting the recovery of motor and cognitive functions after stroke [66].

### 4.1. Circuit Rebuilding: Motor Engrams and Dendritic Dynamics

Post-stroke motor compensation shares fundamental mechanisms with motor learning. It relies on specific circuit rewiring, where NMDAR-mediated LTP consolidates nascent synaptic connections [67,68]. Furthermore, rehabilitation selectively recruits specialized neuronal ensembles within the primary motor cortex (M1), termed motor engram neurons [69]. NMDAR signaling promotes the survival of nascent dendritic spines on these engram neurons and guides their descending axons to form dense spine clusters on specific segments of striatal spiny projection neurons (SPNs). Such structural clustering indicates enhanced cortico-striatal synaptic integration [70,71].

Furthermore, NMDARs bidirectionally regulate dendritic spine dynamics. In addition to driving LTP, specific ligand-binding patterns can engage LTD pathways that promote spine shrinkage [72]. Blocking the immune receptor PirB, which is functionally coupled to NMDAR signaling cascades, mitigates this NMDAR-dependent spine shrinkage [73]. Suppressing such negative plasticity stabilizes newly formed spines, thereby providing the structural substrate for motor skill acquisition and retention. Accordingly, precise regulation of this bidirectional structural plasticity is essential for rewiring damaged cortical networks.

Yet structural remodeling does not occur in isolation. Instead, the permissive or inhibitory characteristics of the surrounding microenvironment determine whether newly formed synapses are stabilized or eliminated.

### 4.2. Rebalancing the Microenvironment for Physiological Plasticity

The physiological plasticity of NMDARs depends on maintaining homeostasis of the synaptic microenvironment. During the chronic recovery phase, endogenous functional remodeling regulates the NMDAR activation environment through intercellular signaling networks [74,75].

It has been demonstrated that interventions such as exercise can upregulate the expression of brain-derived neurotrophic factor (BDNF) in the peri-infarct region, thereby activating the downstream activity-regulated cytoskeleton-associated protein (Arc) [76,77]. The BDNF-Arc signaling axis and postsynaptic NMDARs functionally converge on the regulation of glutamatergic synaptic efficacy [78]. Meanwhile, the reconstruction of internal homeostasis involves adaptive changes in glial cells. An important, but relatively less-studied, mechanism in this glial-neuronal cross-talk involves the astrocytic secretion of thrombospondins (TSPs). TSPs bind to the neuronal α2δ-1 receptor [79]. Although α2δ-1 is classically recognized as an auxiliary subunit of voltage-gated calcium channels, it also directly interacts with NMDARs [80]. Following ischemic injury, this TSP/α2δ-1 signaling axis contributes to the targeting of NMDARs at excitatory synapses and promotes excitatory synaptogenesis [81]. Concomitantly, functional homeostasis is supported by exercise-induced upregulation of glutamate transporters on astrocytes, such as GLT-1 and GLAST [82]. Excess neurotransmitters are cleared from the synaptic cleft, and the extracellular glutamate concentration is lowered by this increase in transporter expression, thereby promoting the phasic activation of NMDARs. The temporal precision for inducing Hebbian plasticity is established by this precise activation pattern [83,84].

Spatial consistency has been identified between gene expression modules underlying NMDAR synaptic signaling and regions showing abnormal distribution of the coefficient of variation of the blood oxygen level-dependent signal (CVBOLD), through integrated transcriptomic and spatial imaging analyses [85].

After IS, diminished NMDAR-mediated excitation in the ipsilesional cortex disrupts interhemispheric balance [86], leading to pathological dominance of the contralesional hemisphere. Excessive transcallosal inhibition further suppresses activity in the injured hemisphere, thereby limiting NMDAR-dependent synaptic plasticity and adaptive remodeling in the perilesional cortex [62,87].

The role of NMDARs in network plasticity has been verified in pharmacological and neurophysiological intervention experiments [88,89]. Although early cell mortality can be reduced by long-term use of non-selective NMDAR channel blockers (e.g., memantine), the macroscopic motor deficit phenotypes of the subject animals are not effectively improved, which may be attributed to the interference with activity-dependent synaptic remodeling [89]. These findings suggest that preserved NMDAR activity is essential for functional recovery after neural injury.

**Figure 2:**
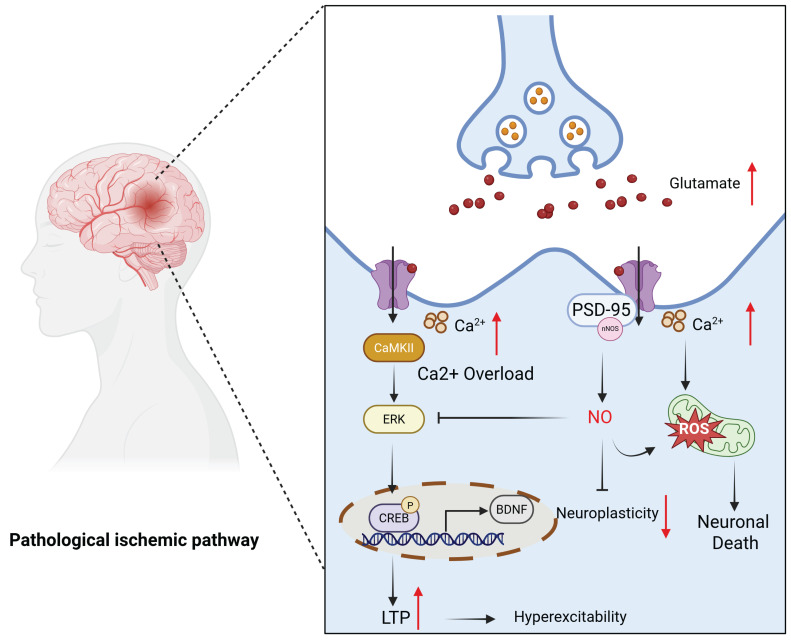
NMDAR-mediated pathological signaling pathways following ischemic stroke. The schematic illustrates the excitotoxic cascade triggered by cerebral ischemia. Cerebral ischemia triggers excessive glutamate release, leading to NMDAR over-activation and intracellular Ca^2+^ overload. (**Left**) The Ca^2+^-dependent CaMKII/ERK/CREB axis modulates BDNF expression; under pathological conditions, aberrant LTP induction contributes to post-stroke hyperexcitability. (**Right**) Alternatively, PSD-95/nNOS coupling promotes NO production, which inhibits the ERK pathway and stimulates mitochondrial ROS generation. These synergistic processes lead to impaired neuroplasticity and accelerate neuronal death.

The loss of physiological NMDAR plasticity represents a central barrier to post-stroke network reorganization. This is particularly evident because changes in cortical excitability induced by transcranial direct current stimulation (tDCS) and paired associative stimulation (PAS) depend on the normal gating function of NMDARs [90,91]. Under conditions of impaired plasticity, physiological receptor function can be restored by targeted intervention with D-cycloserine, a partial agonist at the glycine-binding site of NMDARs. Through this modulation, dendritic arborization is promoted and synaptic density increases within the peri-infarct cortex, while reducing glial fibrillary acidic protein (GFAP)-positive reactive astrogliosis [92,93]. Consequently, brain stimulation-induced plasticity can be reinstated, which correlates with restored LTP-like plasticity and improved motor sequence learning. These findings suggest that functional recovery after stroke depends on the restoration of physiological spatiotemporal regulation of NMDAR activity rather than its indiscriminate enhancement (Figure 2).

## 5. Therapeutic Potential and Clinical Translation

Functional recovery after IS is limited not only by damage during the acute phase but also by abnormal neuroplasticity during the subacute and chronic phases. The role of NMDARs in synaptic transmission and network reorganization, as previously described, is considered a critical translational target for intervening in post-stroke cognitive, emotional, and motor complications. Current pharmacological and neuromodulatory research is shifting from non-selective global receptor blockade toward precision interventions targeting specific receptor subunits and protein–protein interaction interfaces. However, as most mechanistic findings are based on animal models or in vitro experiments, their translation to clinical practice still requires extremely cautious evaluation [94,95].

### 5.1. Cognitive and Memory Impairment: Biomarkers and Precision Subtype Modulation

Post-stroke cognitive impairment (PSCI) and long-term memory deficits represent core complications affecting functional independence. Autoimmune responses directed against synaptic proteins can be triggered by ischemia-induced blood–brain barrier (BBB) disruption. The emergence of serum NMDAR1 autoantibodies (NMDAR1-abs) during the acute phase of stroke has been significantly correlated with an increased risk of memory impairment and broader neuropsychiatric outcomes at 12 months post-stroke [96,97]. The clustering and internalization of synaptic surface NMDARs can be mediated by these autoantibodies, resembling the pathological alterations observed in anti-NMDAR encephalitis models. This indicates that certain cognitive deficits may be fundamentally driven by the immune-mediated impairment of structural plasticity [98,99]. The improvement of vascular dementia is dependent on the optimization of the synaptic signal-to-noise ratio. Modest clinical benefits in such degenerative pathologies have been demonstrated by the low-affinity uncompetitive antagonist memantine [100]. New-generation memantine derivatives, such as fluoroethylnormemantine (FENM), have been developed to optimize therapeutic efficacy and expand clinical indications. Preclinical studies indicate that FENM effectively promotes extinction learning and reverses stress-induced adverse behaviors, while avoiding the motor side effects of traditional blockers [101,102].

Furthermore, pharmacological precision can be improved by positive allosteric modulators (PAMs) targeting specific subunits. For PAMs targeting the ATD or LBD of GluN2A, channel open time can be prolonged upon endogenous transmitter binding, and relative selectivity over GluN2B-containing receptors is exhibited, whereby hippocampal synaptic transmission and spatial memory are enhanced [103,104]. Within rehabilitative paradigms, improvements in cognitive behavior have been shown to be accompanied by increased synaptic insertion of AMPA receptors in the hippocampus following combined treatment with environmental enrichment and brain protein hydrolysate. In contrast, total NMDAR subunit levels remain unchanged [105,106]. Consequently, cognitive recovery is primarily dictated by the precise regulation of receptor trafficking and downstream signaling, rather than by simple upregulation of total receptor abundance.

### 5.2. Post-Stroke Depression: Extrasynaptic Mechanisms and Metabolic Reprogramming

Post-stroke depression (PSD) is associated with psychological stress and is intricately linked to ischemia-induced abnormalities within glutamatergic networks. According to the glutamatergic hypothesis of depression, extrasynaptic NMDARs within emotion-regulating networks are overactivated by ischemia-induced glutamate spillover; this spatially restricted overactivation mediates early neurotoxicity and also drives the sustained suppression of synaptogenesis during the chronic phase via inhibition of CREB and BDNF signaling as well [26,107].

In rapid antidepressant mechanisms, tonic NMDAR signaling driven by spontaneous glutamate release is blocked by NMDAR antagonists such as ketamine [108]. Relief of phosphorylation-mediated inhibition of eukaryotic elongation factor 2 (eEF2) rapidly enhances local synaptic translation of BDNF, thereby promoting dendritic spinogenesis and structural plasticity in the prefrontal cortex and hippocampus [109]. mGluR5 signaling may further contribute to this process by regulating local synaptic Ca2+ microdomains and integrating AMPAR-mTOR-BDNF-associated pathways to promote synaptic plasticity-related protein expression [109].

The reprogramming of endogenous metabolic networks is recognized as another core mechanism driving PSD. Tryptophan metabolism is shifted toward the kynurenine pathway by stroke-induced neuroinflammation, leading to the abnormal accumulation of the endogenous NMDAR agonist quinolinic acid (QUIN) and the relative depletion of the endogenous antagonist kynurenic acid (KYNA) [110,111]. Therefore, the restoration of the KYNA/QUIN ratio through targeted modulation of key enzymes within this pathway is a potential translational strategy to indirectly regulate receptor activity and ameliorate affective disorders.

### 5.3. Preserving Neurovascular Function and Decoupling Lethal Complexes

Cortical spreading depolarization (CSD) is a critical event depleting local energy metabolism and driving microvascular spasm, the initiation and propagation of which are critically mediated by NMDAR activation [112,113]. Memantine exerts long-term protective effects on neurovascular function by reducing CSD burden and improving local perfusion parameters [114].

To block excitotoxicity while maximally preserving physiological receptor functions, PPIs can be one of the emerging translational directions. The transient receptor potential channel TRPM4 is a key transmembrane partner that mediates toxicity signals [115]. By disrupting the NMDAR-TRPM4 heterocomplex with specific interface inhibitors, intracellular sodium and calcium overload are effectively reduced, and infarct volume is decreased, while normal LTP and cognitive functions are largely preserved [116,117].

NMDARs have been widely shown to form macromolecular heteroreceptor complexes with G protein-coupled receptors (GPCRs) [118]. Accordingly, targeting GPCR components to indirectly modulate NMDAR activity may represent a promising strategy for developing neuroprotective agents with improved safety profiles [119].

### 5.4. Motor Recovery: Excitation–Inhibition Imbalance and NMDAR-Dependent Network Plasticity

Following a stroke, excitatory synaptic transmission within the primary motor cortex undergoes profound remodeling. This results in a persistent E/I imbalance within the peri-infarct region [119], which serves as a critical pathophysiological driver of motor dysfunction [120]. Electrophysiological evidence indicates that ischemia leads to prolonged channel open times and increased open probability of both NMDA and AMPA receptors in pyramidal neurons. These changes reflect an amplification of synaptic glutamatergic signaling, resulting in enhanced NMDA-receptor- and AMPA-receptor-mediated synaptic responses [121]. These alterations in synaptic kinetics not only reflect an exacerbation of excitotoxic processes during the acute phase but may also exert lasting effects on the integration of sensorimotor networks and subsequent functional reorganization. Modulation of NMDAR-mediated excitatory transmission, which consequently influences synaptic plasticity and neural network remodeling, may represent a fundamental mechanism driving motor recovery post-stroke. Evidence suggests that modulation of NMDAR signaling plays an important role in improving sensorimotor function [122]. In particular, enhancing glutamatergic synaptic transmission within the cortex has been associated with improved motor recovery following stroke [123].

However, direct evidence remains limited, and findings are inconsistent. For instance, Cherry et al. investigated the combination of D-cycloserine with motor training but did not observe significant improvements in motor outcomes [124]. In contrast, low-affinity NMDAR antagonists with rapid dissociation kinetics, such as amantadine and memantine, have demonstrated clinical benefits in other neurological conditions, including Parkinson’s disease-related motor and cognitive impairments [125,126]. Nevertheless, direct evidence supporting their efficacy in post-stroke motor recovery remains scarce [127].

Valdés et al. reported that memantine may enhance motor learning during the rehabilitation phase [128]. But large-scale clinical trials are still required to establish its efficacy and safety for post-stroke motor recovery [129]. Existing studies have predominantly focused on acute neuroprotection, such as reducing excitotoxicity and infarct burden. NMDAR-targeted interventions for motor recovery in the chronic phase remain relatively underexplored [12]. While extensive efforts have been dedicated to acute-phase neuroprotection aimed at reducing excitotoxicity and infarct burden, NMDAR-targeted research directed toward functional motor recovery in the chronic phase remains significantly underexplored. Notably, motor dysfunction in the chronic phase is not solely determined by neuronal survival and functional preservation during the acute phase. However, it is also closely associated with persistent E/I imbalance and aberrant network activity [130]. Fine-tuned modulation of NMDARs through subunit-specific regulation of GluN2A/GluN2B and spatial control of synaptic and extrasynaptic receptor distribution, together with restoration of E/I balance via the GABAergic system, may represent a promising strategy for promoting motor recovery. Future studies should focus on phase-specific and subtype-selective NMDAR modulation and integrate these approaches with rehabilitation paradigms to systematically evaluate their efficacy and safety in post-stroke motor recovery [131].

### 5.5. Spasticity: NMDAR-Driven Hyperexcitability and Maladaptive Plasticity

Post-stroke spasticity fundamentally arises from the impairment of descending supraspinal inhibitory signals, which unleashes the hyperexcitability of the reticulospinal tract and spinal motor neurons [132]. This abnormal firing ultimately manifests as increased muscle tone and involuntary muscle contractions [133]. Although current clinical interventions such as botulinum toxin injections and GABAergic agonists can temporarily mask this state of hyperexcitability, their therapeutic effects are often transient and accompanied by adverse side effects [134,135,136]. Furthermore, direct targeting strategies aimed at KCC2 remain in the exploratory phase regarding pharmaceutical translation; their long-term safety and clinical feasibility require further validation [137]. NMDAR-driven excitatory toxicity acts as a central molecular catalyst in driving this maladaptive hyperexcitability [138,139]. As ionotropic glutamate receptors are highly sensitive to Ca^2+^ signaling, NMDARs are widely expressed in spinal motor neurons and act in concert with KCC2 and GABA_A receptors to regulate motor neuron excitability [140]. After a stroke, excessive activation of NMDARs may impair KCC2 expression or function through PP1- and calpain-dependent protein degradation pathways, thereby weakening GABAergic inhibition and further exacerbating motor neuron hyperexcitability [141]. Given the central role of glutamatergic signaling in excitatory synaptic transmission, targeting NMDAR-mediated excitatory drive may provide a complementary strategy to inhibition-enhancing approaches for restoring E/I balance. Indeed, the potential involvement of NMDARs in spasticity after spinal cord injury has received increasing attention.

Further investigation of NMDAR-targeted interventions in both stroke models and clinical populations holds considerable translational promise. Existing evidence suggests that modulation of NMDAR-related pathways may exert central regulatory effects. For example, He et al. demonstrated that acupuncture modulated the spinal NMDAR–PP1/Calpain1–KCC2 pathway, effectively suppressing motor neuron hyperexcitability, alleviating spasticity in MCAO rats, and promoting motor functional recovery [141]. Nevertheless, its precise mechanisms of action, durability, and long-term safety remain uncertain and require further validation in stroke-relevant preclinical and clinical studies. From a mechanistic perspective, coordinated modulation of glutamatergic and GABAergic signaling may provide a more comprehensive strategy for restoring E/I balance by attenuating aberrant excitatory transmission while reinforcing impaired inhibitory input. The clinical value of NMDAR modulation in post-stroke spasticity has yet to be established, particularly with respect to treatment timing, dosing strategy, and long-term safety.

## 6. Historical Lessons and Future Directions of NMDAR-Targeted Therapy in Ischemic Stroke

Based on their central roles in the physiological and pathological mechanisms discussed above, NMDARs have long been considered among the most attractive targets for neuroprotective drug development in ischemic stroke. In theory, blocking aberrant NMDAR-mediated excitatory transmission could reduce post-ischemic neuronal death and limit the progression of stroke-related pathological injury and complications. Preclinical studies suggested neuroprotective potential for classical NMDAR antagonists, including Aptiganel, Selfotel, and Gavestinel/GV150526. Aptiganel significantly reduced final infarct volume in a rat model of permanent MCAO. Selfotel attenuated infarct area and post-ischemic hypermetabolic responses, and Gavestinel/GV150526 demonstrated an extended therapeutic window with evidence of cortical functional preservation in experimental MCAO models [142,143,144]. However, subsequent clinical trials yielded disappointing results. Although these agents reduced anatomical injury in animal models, they failed to consistently improve functional outcomes in patients with acute stroke. Some compounds were also associated with mortality imbalance, psychiatric adverse events, hypertension, or dose-limiting toxicity [145,146,147,148]. These findings indicate that the clinical translation of NMDAR-targeted therapy in ischemic stroke remains challenging and requires further investigation.

The failure of clinical translation is unlikely to be attributable to a single factor. Rather, it reflects multiple challenges, including therapeutic-window mismatch, insufficient pharmacological selectivity, dose-related toxicity, and the biological demands of functional recovery (see Supplementary Table S1). In preclinical studies, NMDAR antagonists were often administered before ischemia or within minutes to tens of minutes after ischemic onset. In contrast, in human clinical trials, treatment was typically initiated several hours after stroke onset. In rodents, the glutamate-mediated excitotoxic window may be largely restricted to the first 10–30 min after injury. In emergency clinical settings, however, the peak of acute glutamate toxicity may have already passed by the time treatment is delivered. NMDAR blockade at this stage may therefore provide limited neuroprotection and may even interfere with neuronal survival and repair-related signaling [9]. In addition, early potent NMDAR antagonists were frequently constrained by dose-limiting toxicity. Although Aptiganel showed robust neuroprotective effects in animal models, human studies reported hypertension, sedation, and other central nervous system adverse events [141,149]. Similarly, the clinical use of Selfotel was limited by safety concerns, including hallucinations, agitation, confusion, delirium, and mortality imbalance [144,147]. On the other hand, NMDAR signaling is not restricted to excitotoxic injury, but also contributes to synaptic transmission, neuronal survival, activity-dependent plasticity, and long-term functional reorganization. Accordingly, non-selective NMDAR blockade may attenuate excitotoxic damage during the acute phase while suppressing physiological plasticity signals required for subacute and chronic recovery. Consistent with this concern, memantine has been shown to reduce ischemic injury at low doses but exacerbate brain damage at higher doses, highlighting the dose-dependent bidirectionality of NMDAR modulation [150]. In parallel, aberrantly enhanced post-stroke neurogenesis may worsen cognitive impairment, indicating that increased plasticity is not inherently beneficial and requires phase- and network-specific regulation [151]. Therefore, the central challenge in NMDAR-targeted drug development is not simply whether NMDARs should be inhibited, but how pathological excitotoxicity can be limited while preserving, or even facilitating, physiological NMDAR signaling required for functional recovery.

Direct NMDAR blockade is frequently hindered by dose-limiting toxicities and the suppression of physiological signaling. To circumvent these limitations, downstream uncoupling strategies have emerged as a promising alternative. Nerinetide (NA-1/Tat-NR2B9c) represents the most extensively investigated agent within this new paradigm [152]. Unlike conventional antagonists, nerinetide does not directly obstruct the receptor pore but instead selectively disrupts the interaction between the GluN2B subunit and PSD-95. This targeted molecular intervention effectively uncouples NMDARs from downstream neurotoxic signaling pathways, particularly nNOS activation, while preserving physiological synaptic transmission [153]. Preclinical investigations demonstrate the robust neuroprotective profile of nerinetide. It protects the neurovascular unit by stabilizing the BBB and attenuating pericyte-mediated no-reflow phenomena [154,155]. Moreover, its ability to restrict infarct growth and preserve motor function in gyrencephalic non-human primates successfully bridges the translational gap toward clinical application [156].

Despite this strong preclinical rationale, the clinical translation of Nerinetide has been challenged by complex pharmacokinetic limitations and spatiotemporal barriers. In the pivotal Phase III ESCAPE-NA1 trial, no statistically significant functional benefit was observed in the overall study population [157]. Subsequent analyses revealed that alteplase-induced plasmin generation rapidly cleaved the nerinetide peptide and abolished its therapeutic activity, ultimately driving this unanticipated neutral outcome [158]. Post hoc analyses and the ESCAPE-NEXT trial subsequently exposed a profound temporal mismatch between the ultra-short neuroprotective window and the rapid reperfusion kinetics of modern endovascular thrombectomy (EVT) [157,159]. These translational challenges have prompted a critical reassessment of existing preclinical paradigms. Recent comparative studies employing severe permanent ischemia models or delayed treatment paradigms, such as administration 2 h post-onset, demonstrated that the efficacy of isolated PSD-95 blockade declines rapidly over time [160]. In contrast, multi-target pleiotropic compounds, such as poly-arginine R18, have exhibited a broader therapeutic window and superior long-term neurobehavioral protection under similarly stringent experimental conditions [161].

Unlike early potent NMDAR antagonists such as Aptiganel and Selfotel, memantine is a low-affinity, uncompetitive open-channel antagonist that may preferentially suppress pathologically overactivated NMDARs while preserving a degree of physiological synaptic signaling. Representative preclinical and clinical evidence for memantine and other NMDAR modulators is summarized in Supplementary Table S2.

In acute ischemic models, multiple animal studies have shown that memantine can reduce infarct volume, improve behavioral outcomes, preserve BBB integrity, and enhance the safety of delayed thrombolysis [162,163,164]. In addition, combination strategies pairing NMDAR antagonists with agents targeting complementary pathological processes may provide stronger neuroprotective effects. For example, Jung et al. showed that memantine combined with sodium nitrite extended the therapeutic window and attenuated the potential toxicity associated with NO burst [165]. Culmsee et al. found that memantine combined with clenbuterol enhanced neuroprotection and extended the effective intervention window [166]. Kilic et al. further showed that memantine combined with melatonin produced stronger anti-ischemic effects than monotherapy [167]. Therefore, the application of NMDAR antagonists should not be limited to a single excitotoxic pathway, but should establish more refined combination strategies across pathological excitotoxicity, oxidative stress, vascular barrier injury, and metabolic support.

Therapeutic strategies targeting NMDARs should also consider their potential impact on long-term functional recovery after stroke. Lessons from previous NMDAR antagonist studies indicate that anatomical neuroprotection does not necessarily translate into meaningful improvements in motor, language, cognitive, or affective outcomes. Functional recovery after stroke depends on activity-dependent synaptic plasticity, neural network reorganization, and rehabilitation-induced functional remodeling, processes that are closely linked to NMDAR signaling. Pigretti et al. reported that memantine combined with constraint-induced aphasia therapy improved aphasia quotient, naming ability, spontaneous speech, and repetition in patients with chronic post-stroke aphasia, suggesting that NMDAR modulation may enhance experience-dependent cortical plasticity when paired with rehabilitation training [168]. Zhang et al. reported that memantine-preconditioned extracellular vesicles derived from human umbilical cord mesenchymal stem cells improved post-stroke motor function and spatial memory recovery more effectively than conventional EVs, suggesting that NMDAR-related preconditioning may enhance the functional benefits of regenerative repair strategies [169]. Nevertheless, this line of research remains underdeveloped. Chronic-phase NMDAR modulation should therefore be considered a promising yet underexplored translational direction.

Future studies should integrate disease stage, receptor localization, subunit-specific features, drug delivery capacity, and rehabilitation paradigms to determine whether NMDAR modulation can translate reductions in anatomical injury into meaningful improvements in functional outcomes. This perspective may help address key limitations of previous studies, including therapeutic-window mismatch, non-selective inhibition, dose-related toxicity, and insufficient functional benefit.

## 7. Discussion

Despite extensive research into neuroprotection, NMDAR-targeted therapies for IS have largely failed to demonstrate consistent clinical benefit. Traditional approaches have heavily relied on “anatomical salvaging”—aiming to prevent acute neuronal death by blunting excitotoxic Ca^2+^ overload. However, mounting evidence indicates that mere structural survival does not guarantee functional recovery. We propose that early disruption of neuronal activity, driven by aberrant NMDAR signaling, may represent a unifying axis linking acute injury, maladaptive plasticity, and long-term functional deficits. This perspective highlights the importance of restoring physiological network activity in addition to preserving neuronal survival.

During the acute phase of IS, massive glutamate efflux and the extrasynaptic redistribution of NMDARs do more than trigger cell death cascades; they introduce substantial electrophysiological noise in surviving neurons, manifesting as increased firing variability and network desynchronization. This is supported by recent in vivo imaging and electrophysiological studies demonstrating that early alterations in cortical excitability disrupt interhemispheric balance well before structural atrophy becomes apparent [59]. Such early disturbances may interfere with the emergence of a post-stroke sensitive period for plasticity, thereby constraining subsequent functional reorganization.

When synaptic remodeling unfolds under these distorted conditions, it promotes maladaptive plasticity. This state is characterized by the stabilization of aberrant connectivity and is further reinforced by non-ionotropic NMDAR signaling and pathological protein interactions, notably the NMDAR–TRPM4 complex. Consequently, these unresolved early activity disturbances may limit the effective consolidation of motor engrams and constrain motor recovery.

PSCI and PSD are traditionally managed as distinct downstream sequelae. From a systems neuroscience perspective, however, persistent E/I imbalance may serve as a convergent systems-level readout integrating neuroinflammation, metabolic dysregulation, and aberrant synaptic remodeling. Sustained NMDAR dysregulation may suppress critical neurotrophic signaling pathways, such as the BDNF–Arc axis, and is associated with alterations in tryptophan metabolism. At the network level, these persistent dynamic abnormalities have been linked to cognitive and affective dysfunction. Consistent with this view, clinical neuroimaging studies have shown that the extent of network connectivity disruption during the subacute phase predicts long-term neuropsychiatric outcomes [62]. Accordingly, PSCI and PSD may be better conceptualized as delayed functional consequences of impaired early network reorganization rather than entirely independent disease entities.

Addressing these translational challenges will likely require approaches that more precisely modulate NMDAR function. Rather than broadly suppressing receptor activity, emerging strategies aim to dissociate neurotoxic signaling from physiological receptor functions involved in maintaining excitation–inhibition balance. It is critical to distinguish between limiting acute excitotoxicity and supporting recovery-related plasticity. In the acute phase of stroke, moderate suppression of pathological NMDAR overactivation may help mitigate Ca^2+^ overload, BBB disruption, and neurovascular unit injury. In the subacute and chronic recovery phases, by contrast, sustained or excessive inhibition of NMDAR signaling may interfere with synaptic plasticity, motor learning, language recovery, and network reorganization.

Future studies should therefore evaluate NMDAR modulation across distinct disease stages and distinguish the functional roles of synaptic and extrasynaptic receptor signaling. In particular, the roles of GluN2A and GluN2B should not be simply categorized as protective or damaging subunits. Although GluN2B may contribute to excitotoxic processes during the acute phase, it may also support synaptic remodeling and functional network reconstruction during recovery. Pharmacological tools, including positive allosteric modulators and subunit-selective interventions, may enable more selective control of NMDAR-dependent processes.

Importantly, considerable clinical promise may lie in synergistic paradigms that integrate pharmacological priming with system-level neuromodulation. Biomarkers derived from neuroimaging or continuous electrophysiological monitoring may help identify individualized therapeutic windows. Within these windows, pharmacological agents could lower the threshold for physiological plasticity, creating a permissive environment for targeted non-invasive brain stimulation and task-specific rehabilitation to guide adaptive network reorganization. The feasibility of this approach is supported by emerging evidence suggesting that partial NMDAR modulation can reinstate neuroplastic responses to transcranial stimulation [92,93].

Given the persistent challenges regarding patient heterogeneity, optimal treatment timing, and the need for reliable biomarkers, successfully navigating the translational divide logically dictates a shift toward the precise, spatiotemporal modulation of NMDAR signaling to ultimately drive functional recovery and minimize long-term sequelae.

## 8. Conclusions

The pathophysiology of the nervous system following IS undergoes a dynamic evolution, progressing from acute excitotoxic injury to structural remodeling and functional compensation during the subacute and chronic phases. NMDARs play a pivotal role throughout this continuum. While their overactivation during the acute phase contributes to neuronal injury, in later stages, they support recovery-related processes, including synaptic plasticity, neural circuit reconstruction, and network reorganization. Notably, the functional outcomes of NMDAR signaling are highly context dependent, shaped by receptor subunit composition, subcellular localization, cell type, and disease stage. Consequently, multiple stages of post-stroke pathophysiology are profoundly modulated by their multifaceted roles, establishing them as a critical molecular target for stroke intervention.

## Data Availability

No new data were created or analyzed in this study.

## Supplementary Materials

The following supporting information can be downloaded at https://www.mdpi.com/article/10.3390/biom16060770/s1. Table S1: Clinical trial outcomes of classic NMDA receptor antagonist drugs for ischemic stroke treatment [9,142,143,144,145,146,147,149,152,153,156,157,158,159,170,171,172,173,174,175,176,177,178,179,180,181]; Table S2: Mechanistic exploration and expanded application of novel NMDA-related neuroprotective therapies [47,150,151,154,155,156,160,161,162,163,164,165,166,167,169,182,183,184,185,186,187,188,189,190,191,192,193,194,195,196,197,198,199,200,201,202,203,204,205].

## Abbreviations

The following abbreviations are used in this manuscript:

IS	Ischemic stroke
NMDAR	N-methyl-D-aspartate Receptor
iGluRs	Ionotropic glutamate receptors
AMPA	α-amino-3-hydroxy-5-methyl-4-isoxazolepropionic acid
KA	Kainate
ATD	Amino-terminal domain
LBD	Ligand-binding domain
TMD	Transmembrane domain
CTD	Carboxyl-terminal domain
LTP	Long-term potentiation
CaM	Calmodulin
CaMKII	Ca^2+^/calmodulin-dependent protein kinase II
PSD-95	Postsynaptic density-95
MAGUK	Membrane-associated guanylate kinase
nNOS	Neuronal nitric oxide synthase
CREB	cAMP response element-binding protein
PPIs	Protein–protein interaction
RNS	Reactive nitrogen species
DAPK1	Death-associated protein kinase 1
TRPM4	Transient receptor potential melastatin 4
NO	Nitric oxide
ROS	Reactive oxygen species
BDNF	Brain-derived neurotrophic factor
Arc	Activity-regulated cytoskeleton-associated protein
GLT-1	Glutamate transporter-1
GLAST	Glutamate-aspartate transporter
CV_BOLD_	Coefficient of variation of the blood oxygen level-dependent signal
tDCS	Transcranial direct current stimulation
PAS	Paired associative stimulation
GFAP	Glial fibrillary acidic protein
PSCI	Post-stroke cognitive impairment
NMDAR1-abs	NMDAR1 autoantibodies
BBB	Blood–brain barrier
PAMs	Positive allosteric modulators
PSD	Post-stroke depression
eEF2	Eukaryotic elongation factor 2
mGluR5	Metabotropic glutamate receptor 5
mTOR	Mammalian target of rapamycin
QUIN	Quinolinic acid
KYNA	Kynurenic acid
CSD	Cortical spreading depolarization
GPCRs	G protein-coupled receptors
MTD	Maximum Tolerated Dose

## References

[1] Feske S.K. (2021). Ischemic Stroke. Am. J. Med..

[2] Weaver N.A., Kuijf H.J., Aben H.P., Abrigo J., Bae H.J., Barbay M., Best J.G., Bordet R., Chappell F.M., Chen C. (2021). Strategic infarct locations for post-stroke cognitive impairment: A pooled analysis of individual patient data from 12 acute ischaemic stroke cohorts. Lancet Neurol..

[3] Zhang Z., Wang M., Gill D., Liu X., Zhu W. (2024). Association of Genetically Predicted Anxiety and Depression With Functional Outcome After Ischemic Stroke: A Mendelian Randomization Study. Neurology.

[4] Lai K., Pritišanac I., Liu Z.Q., Liu H.W., Gong L.N., Li M.X., Lu J.F., Qi X., Xu T.L., Forman-Kay J. (2024). Glutamate acts on acid-sensing ion channels to worsen ischaemic brain injury. Nature.

[5] Duan J.J., Jiang B., Yin W., Lin Y., Yan G.M., Lu W. (2025). Cell-autonomous GABAARs are essential for NMDAR-mediated synaptic transmission, LTP, and spatial memory. EMBO Rep..

[6] Paoletti P., Bellone C., Zhou Q. (2013). NMDA receptor subunit diversity: Impact on receptor properties, synaptic plasticity and disease. Nat. Rev. Neurosci..

[7] Ge Y., Chen W., Axerio-Cilies P., Wang Y.T. (2020). NMDARs in Cell Survival and Death: Implications in Stroke Pathogenesis and Treatment. Trends Mol. Med..

[8] Lai T.W., Zhang S., Wang Y.T. (2014). Excitotoxicity and stroke: Identifying novel targets for neuroprotection. Prog. Neurobiol..

[9] Ikonomidou C., Turski L. (2002). Why did NMDA receptor antagonists fail clinical trials for stroke and traumatic brain injury?. Lancet Neurol..

[10] Hansen K.B., Wollmuth L.P., Bowie D., Furukawa H., Menniti F.S., Sobolevsky A.I., Swanson G.T., Swanger S.A., Greger I.H., Nakagawa T. (2021). Structure, Function, and Pharmacology of Glutamate Receptor Ion Channels. Pharmacol. Rev..

[11] Reiner A., Levitz J. (2018). Glutamatergic Signaling in the Central Nervous System: Ionotropic and Metabotropic Receptors in Concert. Neuron.

[12] Gong H., Xu X., Talifu Z., Zhang C.J., Sun Y.Z., Yue Z.M., Rao J.S., Du L.J., Du X.X. (2025). Prospects and challenges in NMDAR signaling in spinal cord injury recovery and neural circuit remodeling. Regen. Ther..

[13] Mony L., Paoletti P. (2023). Mechanisms of NMDA receptor regulation. Curr. Opin. Neurobiol..

[14] Kang H., Epstein M., Banke T.G., Perszyk R., Simorowski N., Paladugu S., Liotta D.C., Traynelis S.F., Furukawa H. (2025). Structural basis for channel gating and blockade in tri-heteromeric GluN1-2B-2D NMDA receptor. Neuron.

[15] Schrattenholz A., Soskic V. (2006). NMDA receptors are not alone: Dynamic regulation of NMDA receptor structure and function by neuregulins and transient cholesterol-rich membrane domains leads to disease-specific nuances of glutamate-signalling. Curr. Top. Med. Chem..

[16] Khatri A., Burger P.B., Swanger S.A., Hansen K.B., Zimmerman S., Karakas E., Liotta D.C., Furukawa H., Snyder J.P., Traynelis S.F. (2014). Structural determinants and mechanism of action of a GluN2C-selective NMDA receptor positive allosteric modulator. Mol. Pharmacol..

[17] Karakas E., Furukawa H. (2014). Crystal structure of a heterotetrameric NMDA receptor ion channel. Science.

[18] Chou T.H., Epstein M., Fritzemeier R.G., Akins N.S., Paladugu S., Ullman E.Z., Liotta D.C., Traynelis S.F., Furukawa H. (2024). Molecular mechanism of ligand gating and opening of NMDA receptor. Nature.

[19] Monyer H., Sprengel R., Schoepfer R., Herb A., Higuchi M., Lomeli H., Burnashev N., Sakmann B., Seeburg P.H. (1992). Heteromeric NMDA receptors: Molecular and functional distinction of subtypes. Science.

[20] Abbott J.A., Kim J., Liu B., Popescu G.K., Gouaux E., Jalali-Yazdi F. (2025). Cryo-EM snapshots of NMDA receptor activation illuminate sequential rearrangements. Sci. Adv..

[21] Tian M., Stroebel D., Piot L., David M., Ye S., Paoletti P. (2021). GluN2A and GluN2B NMDA receptors use distinct allosteric routes. Nat. Commun..

[22] Hansen K.B., Yi F., Perszyk R.E., Furukawa H., Wollmuth L.P., Gibb A.J., Traynelis S.F. (2018). Structure, function, and allosteric modulation of NMDA receptors. J. Gen. Physiol..

[23] Peoples R.W., Ren H. (2022). Effects of ethanol on GluN1/GluN2A and GluN1/GluN2B NMDA receptor-ion channel gating kinetics. Alcohol. Clin. Exp. Res..

[24] Amin J.B., He M., Prasad R., Leng X., Zhou H.X., Wollmuth L.P. (2023). Two gates mediate NMDA receptor activity and are under subunit-specific regulation. Nat. Commun..

[25] Lussier M.P., Sanz-Clemente A., Roche K.W. (2015). Dynamic Regulation of N-Methyl-d-aspartate (NMDA) and α-Amino-3-hydroxy-5-methyl-4-isoxazolepropionic Acid (AMPA) Receptors by Posttranslational Modifications. J. Biol. Chem..

[26] Hardingham G.E., Bading H. (2010). Synaptic versus extrasynaptic NMDA receptor signalling: Implications for neurodegenerative disorders. Nat. Rev. Neurosci..

[27] Cull-Candy S., Brickley S., Farrant M. (2001). NMDA receptor subunits: Diversity, development and disease. Curr. Opin. Neurobiol..

[28] Keith R.E., Wild G.A., Keith M.J., Chen D., Pack S., Dumas T.C. (2024). Individual NMDA receptor GluN2 subunit signaling domains differentially regulate the postnatal maturation of hippocampal excitatory synaptic transmission and plasticity but not dendritic morphology. Synapse.

[29] Gray J.A., Zito K., Hell J.W. (2016). Non-ionotropic signaling by the NMDA receptor: Controversy and opportunity. F1000Research.

[30] Paoletti P., Neyton J. (2007). NMDA receptor subunits: Function and pharmacology. Curr. Opin. Pharmacol..

[31] Hell J.W. (2023). Binding of CaMKII to the NMDA receptor is sufficient for long-term potentiation. Sci. Signal.

[32] Nicoll R.A., Schulman H. (2023). Synaptic memory and CaMKII. Physiol. Rev..

[33] Barnes S.A., Thomazeau A., Finnie P.S.B., Heinrich M.J., Heynen A.J., Komiyama N.H., Grant S.G.N., Menniti F.S., Osterweil E.K., Bear M.F. (2025). Non-ionotropic signaling through the NMDA receptor GluN2B carboxy-terminal domain drives dendritic spine plasticity and reverses fragile X phenotypes. Cell Rep..

[34] Le A.A., Lauterborn J.C., Jia Y., Cox C.D., Lynch G., Gall C.M. (2024). Metabotropic NMDAR Signaling Contributes to Sex Differences in Synaptic Plasticity and Episodic Memory. J. Neurosci..

[35] Christopherson K.S., Hillier B.J., Lim W.A., Bredt D.S. (1999). PSD-95 assembles a ternary complex with the N-methyl-D-aspartic acid receptor and a bivalent neuronal NO synthase PDZ domain. J. Biol. Chem..

[36] Anwar A., Yameen D., Masood M., Parvez S., Haque M.M. (2025). Neuronal Nitric Oxide Synthase (nNOS) uncoupling in ischemic stroke: Mechanisms of oxidative/nitrosative stress and opportunities for neuroprotection: A review. Int. J. Biol. Macromol..

[37] Martel M.A., Wyllie D.J., Hardingham G.E. (2009). In developing hippocampal neurons, NR2B-containing N-methyl-D-aspartate receptors (NMDARs) can mediate signaling to neuronal survival and synaptic potentiation, as well as neuronal death. Neuroscience.

[38] Shrestha A., Sultana R., Lee C.C., Ogundele O.M. (2019). SK Channel Modulates Synaptic Plasticity by Tuning CaMKIIα/β Dynamics. Front. Synaptic Neurosci..

[39] Larsen M.E., Buonarati O.R., Qian H., Hell J.W., Bayer K.U. (2023). Stimulating β-adrenergic receptors promotes synaptic potentiation by switching CaMKII movement from LTD to LTP mode. J. Biol. Chem..

[40] Ugalde-Triviño L., Díaz-Guerra M. (2021). PSD-95: An Effective Target for Stroke Therapy Using Neuroprotective Peptides. Int. J. Mol. Sci..

[41] Tang A.H., Chen H., Li T.P., Metzbower S.R., MacGillavry H.D., Blanpied T.A. (2016). A trans-synaptic nanocolumn aligns neurotransmitter release to receptors. Nature.

[42] Chaudhari S., Shinde A., Salunke M., Bairagi S., Dhage A., Patel P., Rathod V., Pathare S., Altwaijry N., Khan M.S. (2026). Investigating the anti-Alzheimer potential of biogenic compounds from Zinc15 database as NMDA antagonist: An in-silico approach. J. Mol. Graph. Model..

[43] Al-Diwani A., Theorell J., Zghoul T., Voruganti A., Townsend L., De Giorgi R., Griffin B., Bajorek T., Okai D., Lennox B. (2026). The distinctive psychopathology of NMDAR-antibody encephalitis compared with primary psychoses: An international, multicentre, retrospective phenotypic analysis. Lancet Psychiatry.

[44] Zhang X., Wang D., Cui J., Fan B., Wang F., Lu C. (2025). Neuromodulatory Effects of Arecoline on Anxiety-like Behavior in Mice Exposed to Chronic Unpredictable Mild Stress. Int. J. Mol. Sci..

[45] McEachern E.P., Coley A.A., Yang S.S., Gao W.J. (2020). PSD-95 deficiency alters GABAergic inhibition in the prefrontal cortex. Neuropharmacology.

[46] Dupuis J.P., Nicole O., Groc L. (2023). NMDA receptor functions in health and disease: Old actor, new dimensions. Neuron.

[47] Yu S.P., Jiang M.Q., Shim S.S., Pourkhodadad S., Wei L. (2023). Extrasynaptic NMDA receptors in acute and chronic excitotoxicity: Implications for preventive treatments of ischemic stroke and late-onset Alzheimer’s disease. Mol. Neurodegener..

[48] Shen Z., Xiang M., Chen C., Ding F., Wang Y., Shang C., Xin L., Zhang Y., Cui X. (2022). Glutamate excitotoxicity: Potential therapeutic target for ischemic stroke. Biomed. Pharmacother..

[49] Bell K.F., Bent R.J., Meese-Tamuri S., Ali A., Forder J.P., Aarts M.M. (2013). Calmodulin kinase IV-dependent CREB activation is required for neuroprotection via NMDA receptor-PSD95 disruption. J. Neurochem..

[50] Li L., Yang R., Sun K., Bai Y., Zhang Z., Zhou L., Qi Z., Qi J., Chen L. (2012). Cerebroside-A provides potent neuroprotection after cerebral ischaemia through reducing glutamate release and Ca^2+^ influx of NMDA receptors. Int. J. Neuropsychopharmacol..

[51] Weilinger N.L., Lohman A.W., Rakai B.D., Ma E.M., Bialecki J., Maslieieva V., Rilea T., Bandet M.V., Ikuta N.T., Scott L. (2016). Metabotropic NMDA receptor signaling couples Src family kinases to pannexin-1 during excitotoxicity. Nat. Neurosci..

[52] Hao L., Wei X., Guo P., Zhang G., Qi S. (2016). Neuroprotective Effects of Inhibiting Fyn S-Nitrosylation on Cerebral Ischemia/Reperfusion-Induced Damage to CA1 Hippocampal Neurons. Int. J. Mol. Sci..

[53] Seelig S., Paramasivam P., Gomez M., Smith A., Miller J.W., Paul S., Poddar R. (2025). GluN2A-NMDAR mediated neuronal NFκB activation plays a key role in exacerbating ischemic brain injury under hyperhomocysteinemic conditions. J. Cereb. Blood Flow. Metab..

[54] Chiu A.M., Wang J., Fiske M.P., Hubalkova P., Barse L., Gray J.A., Sanz-Clemente A. (2019). NMDAR-Activated PP1 Dephosphorylates GluN2B to Modulate NMDAR Synaptic Content. Cell Rep..

[55] Tu W., Xu X., Peng L., Zhong X., Zhang W., Soundarapandian M.M., Balel C., Wang M., Jia N., Zhang W. (2010). DAPK1 interaction with NMDA receptor NR2B subunits mediates brain damage in stroke. Cell.

[56] Stein I.S., Park D.K., Flores J.C., Jahncke J.N., Zito K. (2020). Molecular Mechanisms of Non-ionotropic NMDA Receptor Signaling in Dendritic Spine Shrinkage. J. Neurosci..

[57] Alam J.J., Krakovsky M., Germann U., Levy A. (2020). Continuous administration of a p38α inhibitor during the subacute phase after transient ischemia-induced stroke in the rat promotes dose-dependent functional recovery accompanied by increase in brain BDNF protein level. PLoS ONE.

[58] Oh W.C., Hill T.C., Zito K. (2013). Synapse-specific and size-dependent mechanisms of spine structural plasticity accompanying synaptic weakening. Proc. Natl. Acad. Sci. USA.

[59] Glandorf L., Droux J., Jessen E., Wittmann B., Weber B., Wegener S., Menze B., Leitgeb R., Schillinger D., El Amki M. (2025). In Vivo Network-Level Cerebrovascular Mapping Reveals the Impact of Flow Topology on Capillary Stalls After Stroke. bioRxiv.

[60] Wang T.Y., Feng C., Wang C., Ren C., Zhao Z. (2026). Dynamics of mesoscale brain network during visual discrimination learning revealed by chronic, large-scale single-unit recording. Elife.

[61] Hervella P., Alonso-Alonso M.L., Pérez-Mato M., Rodríguez-Yáñez M., Arias-Rivas S., López-Dequidt I., Pumar J.M., Sobrino T., Campos F., Castillo J. (2022). Surrogate biomarkers of outcome for wake-up ischemic stroke. BMC Neurol..

[62] Ren M., Xu J., Wang W., Shen L., Wang C., Liu H., Chen L., Liu C., Tang Y., Wang J. (2024). Effect of Dual-Site Non-Invasive Brain Stimulation on Upper-Limb Function After Stroke: A Systematic Review and Meta-Analysis. Brain Behav..

[63] Koch P.J., Frey B.M., Backhaus W., Petersen N., Girard G., Wróbel P.P., Braaß H., Bönstrup M., Kunkel Genannt Bode L., Cheng B. (2025). Neurotransmitter-informed connectivity maps and their application for outcome inference after stroke. Brain.

[64] Yamada T., Watanabe T., Sasaki Y. (2024). Plasticity-stability dynamics during post-training processing of learning. Trends Cogn. Sci..

[65] Shepherd J.D., Bear M.F. (2011). New views of Arc, a master regulator of synaptic plasticity. Nat. Neurosci..

[66] Korb E., Finkbeiner S. (2011). Arc in synaptic plasticity: From gene to behavior. Trends Neurosci..

[67] Kessels H.W., Nabavi S., Malinow R. (2013). Metabotropic NMDA receptor function is required for β-amyloid-induced synaptic depression. Proc. Natl. Acad. Sci. USA.

[68] Hu N.W., Klyubin I., Anwyl R., Rowan M.J. (2009). GluN2B subunit-containing NMDA receptor antagonists prevent Abeta-mediated synaptic plasticity disruption in vivo. Proc. Natl. Acad. Sci. USA.

[69] Hwang F.J., Roth R.H., Wu Y.W., Sun Y., Kwon D.K., Liu Y., Ding J.B. (2022). Motor learning selectively strengthens cortical and striatal synapses of motor engram neurons. Neuron.

[70] Miry O., Li J., Chen L. (2020). The Quest for the Hippocampal Memory Engram: From Theories to Experimental Evidence. Front. Behav. Neurosci..

[71] Tonegawa S., Pignatelli M., Roy D.S., Ryan T.J. (2015). Memory engram storage and retrieval. Curr. Opin. Neurobiol..

[72] Albarran E., Raissi A., Jáidar O., Shatz C.J., Ding J.B. (2021). Enhancing motor learning by increasing the stability of newly formed dendritic spines in the motor cortex. Neuron.

[73] Zhao P., Zhang J., Kuai J., Li L., Li X., Feng N., Du H., Li C., Wang Q., Deng B. (2023). TAT-PEP Alleviated Cognitive Impairment by Alleviating Neuronal Mitochondria Damage and Apoptosis After Cerebral Ischemic Reperfusion Injury. Mol. Neurobiol..

[74] Iacobucci G.J., Popescu G.K. (2019). Spatial Coupling Tunes NMDA Receptor Responses via Ca(2+) Diffusion. J. Neurosci..

[75] Yu C., Tyler J.R., Elman I., Blum K., Lewandrowski K.U., Sharafshah A., Gold M.S., Pinhasov A., Thanos P.K. (2025). Exercise-driven modulation of glutamatergic signaling: Mechanisms and clinical implications. Neuroscience.

[76] Sun G.C., Lee Y.J., Lee Y.C., Yu H.F., Wang D.C. (2021). Exercise prevents the impairment of learning and memory in prenatally phthalate-exposed male rats by improving the expression of plasticity-related proteins. Behav. Brain Res..

[77] Belviranlı M., Okudan N. (2018). Exercise Training Protects Against Aging-Induced Cognitive Dysfunction via Activation of the Hippocampal PGC-1α/FNDC5/BDNF Pathway. Neuromol. Med..

[78] Nagahara A.H., Mateling M., Kovacs I., Wang L., Eggert S., Rockenstein E., Koo E.H., Masliah E., Tuszynski M.H. (2013). Early BDNF treatment ameliorates cell loss in the entorhinal cortex of APP transgenic mice. J. Neurosci..

[79] Risher W.C., Eroglu C. (2012). Thrombospondins as key regulators of synaptogenesis in the central nervous system. Matrix Biol..

[80] Catterall W.A. (2000). Structure and regulation of voltage-gated Ca2+ channels. Annu. Rev. Cell Dev. Biol..

[81] Wu T., Chen S.R., Pan H.L., Luo Y. (2023). The α2δ-1-NMDA receptor complex and its potential as a therapeutic target for ischemic stroke. Front. Neurol..

[82] Zhang F., Jia J., Wu Y., Hu Y., Wang Y. (2010). The effect of treadmill training pre-exercise on glutamate receptor expression in rats after cerebral ischemia. Int. J. Mol. Sci..

[83] Feighan K.M., Thakare H.K., Glasgow S.D., Kennedy T.E. (2026). Convergence and divergence of molecular mechanisms in Hebbian and homeostatic plasticity. Front. Synaptic Neurosci..

[84] Zha Y.P., Wang Y.K., Deng Y., Zhang R.W., Tan X., Yuan W.J., Deng X.M., Wang W.Z. (2013). Exercise training lowers the enhanced tonically active glutamatergic input to the rostral ventrolateral medulla in hypertensive rats. CNS Neurosci. Ther..

[85] Guo R., Wang W., Qian R., Ji Y., Li W., Zu M., Li Q., Wu J., Dai W., Xu S. (2026). Decoding neurotransmitter and genetic contributions to abnormal neuronal signal variability in Anti-N-Methyl-D-Aspartate receptor encephalitis: Implications for targeted therapies. Brain Res. Bull..

[86] Yan W., Lin Y., Chen Y.F., Wang Y., Wang J., Zhang M. (2025). Enhancing Neuroplasticity for Post-Stroke Motor Recovery: Mechanisms, Models, and Neurotechnology. IEEE Trans. Neural Syst. Rehabil. Eng..

[87] Xerri C., Zennou-Azogui Y., Sadlaoud K., Sauvajon D. (2014). Interplay between intra- and interhemispheric remodeling of neural networks as a substrate of functional recovery after stroke: Adaptive versus maladaptive reorganization. Neuroscience.

[88] Zrenner C., Ziemann U. (2024). Closed-Loop Brain Stimulation. Biol. Psychiatry.

[89] Ethier C., Gallego J.A., Miller L.E. (2015). Brain-controlled neuromuscular stimulation to drive neural plasticity and functional recovery. Curr. Opin. Neurobiol..

[90] Guidali G., Roncoroni C., Bolognini N. (2021). Paired associative stimulations: Novel tools for interacting with sensory and motor cortical plasticity. Behav. Brain Res..

[91] Tang Z., Liu T., Han K., Liu Y., Su W., Wang R., Zhang H. (2024). The effects of rTMS on motor recovery after stroke: A systematic review of fMRI studies. Neurol. Sci..

[92] Kim H., Kornman P.T., Kweon J., Wassermann E.M., Wright D.L., Li J., Brown J.C. (2024). Combined effects of pharmacological interventions and intermittent theta-burst stimulation on motor sequence learning. bioRxiv.

[93] Adhikari Y., Ma C.G., Chai Z., Jin X. (2023). Preventing development of post-stroke hyperexcitability by optogenetic or pharmacological stimulation of cortical excitatory activity. Neurobiol. Dis..

[94] Capó T., Rebassa J.B., Raïch I., Lillo J., Badia P., Navarro G., Reyes-Resina I. (2025). Future Perspectives of NMDAR in CNS Disorders. Molecules.

[95] Beaurain M., Salabert A.S., Payoux P., Gras E., Talmont F. (2024). NMDA Receptors: Distribution, Role, and Insights into Neuropsychiatric Disorders. Pharmaceuticals.

[96] Arlt F.A., Sperber P.S., von Rennenberg R., Gebert P., Teegen B., Georgakis M.K., Fang R., Dewenter A., Görtler M., Petzold G.C. (2025). Serum anti-NMDA receptor antibodies are linked to memory impairment 12 months after stroke. Mol. Psychiatry.

[97] Deutsch N.R., Worthmann H., Steixner-Kumar A.A., Schuppner R., Grosse G.M., Pan H., Gabriel M.M., Hasse I., van Gemmeren T., Lichtinghagen R. (2021). Autoantibodies against the NMDAR subunit NR1 are associated with neuropsychiatric outcome after ischemic stroke. Brain Behav. Immun..

[98] Planagumà J., Leypoldt F., Mannara F., Gutiérrez-Cuesta J., Martín-García E., Aguilar E., Titulaer M.J., Petit-Pedrol M., Jain A., Balice-Gordon R. (2015). Human N-methyl D-aspartate receptor antibodies alter memory and behaviour in mice. Brain.

[99] Wang H., Xie C., Deng B., Ding J., Li N., Kou Z., Jin M., He J., Wang Q., Wen H. (2024). Structural basis for antibody-mediated NMDA receptor clustering and endocytosis in autoimmune encephalitis. Nat. Struct. Mol. Biol..

[100] McShane R., Westby M.J., Roberts E., Minakaran N., Schneider L., Farrimond L.E., Maayan N., Ware J., Debarros J. (2019). Memantine for dementia. Cochrane Database Syst. Rev..

[101] Chen B.K., Le Pen G., Eckmier A., Rubinstenn G., Jay T.M., Denny C.A. (2021). Fluoroethylnormemantine, A Novel Derivative of Memantine, Facilitates Extinction Learning Without Sensorimotor Deficits. Int. J. Neuropsychopharmacol..

[102] Chen B.K., Luna V.M., Shannon M.E., Hunsberger H.C., Mastrodonato A., Stackmann M., McGowan J.C., Rubinstenn G., Denny C.A. (2021). Fluoroethylnormemantine, a Novel NMDA Receptor Antagonist, for the Prevention and Treatment of Stress-Induced Maladaptive Behavior. Biol. Psychiatry.

[103] Li P., Wang J., Wang M., Chen X., Zhu H., Dong M. (2025). Development of GluN2A NMDA receptor positive allosteric modulators: Recent advances and perspectives. Bioorganic Med. Chem..

[104] Hackos D.H., Lupardus P.J., Grand T., Chen Y., Wang T.M., Reynen P., Gustafson A., Wallweber H.J., Volgraf M., Sellers B.D. (2016). Positive Allosteric Modulators of GluN2A-Containing NMDARs with Distinct Modes of Action and Impacts on Circuit Function. Neuron.

[105] Martínez-Torres N.I., Cárdenas-Bedoya J., Vázquez-Torres B.M., Torres-Mendoza B.M. (2024). Environmental enrichment and cerebrolysin improve motor and cognitive performance in a rat model of stroke, in conjunction with an increase in hippocampal AMPA but not NMDA receptor subunits. Brain Res..

[106] Diering G.H., Huganir R.L. (2018). The AMPA Receptor Code of Synaptic Plasticity. Neuron.

[107] McCarthy D.J., Alexander R., Smith M.A., Pathak S., Kanes S., Lee C.M., Sanacora G. (2012). Glutamate-based depression GBD. Med. Hypotheses.

[108] Autry A.E., Adachi M., Nosyreva E., Na E.S., Los M.F., Cheng P.F., Kavalali E.T., Monteggia L.M. (2011). NMDA receptor blockade at rest triggers rapid behavioural antidepressant responses. Nature.

[109] Moda-Sava R.N., Murdock M.H., Parekh P.K., Fetcho R.N., Huang B.S., Huynh T.N., Witztum J., Shaver D.C., Rosenthal D.L., Alway E.J. (2019). Sustained rescue of prefrontal circuit dysfunction by antidepressant-induced spine formation. Science.

[110] Yao C., Xie D., Zhang Y., Shen Y., Sun P., Ma Z., Li J., Tao J., Fang M. (2026). Tryptophan metabolism and ischemic stroke: An intricate balance. Neural Regen. Res..

[111] Schwarcz R., Bruno J.P., Muchowski P.J., Wu H.Q. (2012). Kynurenines in the mammalian brain: When physiology meets pathology. Nat. Rev. Neurosci..

[112] Mesgari M., Krüger J., Riemer C.T., Khaleghi Ghadiri M., Kovac S., Gorji A. (2017). Gabapentin prevents cortical spreading depolarization-induced disinhibition. Neuroscience.

[113] Miettinen S., Fusco F.R., Yrjänheikki J., Keinänen R., Hirvonen T., Roivainen R., Närhi M., Hökfelt T., Koistinaho J. (1997). Spreading depression and focal brain ischemia induce cyclooxygenase-2 in cortical neurons through N-methyl-D-aspartic acid-receptors and phospholipase A2. Proc. Natl. Acad. Sci. USA.

[114] MacLean M.A., Muradov J.H., Greene R., Van Hameren G., Clarke D.B., Dreier J.P., Okonkwo D.O., Friedman A. (2023). Memantine inhibits cortical spreading depolarization and improves neurovascular function following repetitive traumatic brain injury. Sci. Adv..

[115] Zong P., Legere N., Feng J., Yue L. (2025). TRP Channels in Excitotoxicity. Neuroscientist.

[116] Binkle-Ladisch L., Pironet A., Zaliani A., Alcouffe C., Mensching D., Haferkamp U., Willing A., Woo M.S., Erdmann A., Jessen T. (2024). Identification and development of TRPM4 antagonists to counteract neuronal excitotoxicity. iScience.

[117] Yan J., Bengtson C.P., Buchthal B., Hagenston A.M., Bading H. (2020). Coupling of NMDA receptors and TRPM4 guides discovery of unconventional neuroprotectants. Science.

[118] Franco R., Navarro G. (2023). Neuroprotection afforded by targeting G protein-coupled receptors in heteromers and by heteromer-selective drugs. Front. Pharmacol..

[119] Borroto-Escuela D.O., Tarakanov A.O., Brito I., Fuxe K. (2018). Glutamate heteroreceptor complexes in the brain. Pharmacol. Rep..

[120] Grigoras I.F., Stagg C.J. (2021). Recent advances in the role of excitation-inhibition balance in motor recovery post-stroke. Fac. Rev..

[121] Ikemune K., Mitani A., Namba S., Kataoka K., Arai T. (1999). Functional changes of N-methyl-D-aspartic acid and alpha-amino-3-hydroxy-5-methyl-4-isoxazolepropionate channels in gerbil hippocampal CA1, in relation to postischemic enhancement of glutamate receptor-mediated responses. Neurosci. Lett..

[122] Tang X., Shi J., Lin S., He Z., Cui S., Di W., Chen S., Wu J., Yuan S., Ye Q. (2024). Pyramidal and parvalbumin neurons modulate the process of electroacupuncture stimulation for stroke rehabilitation. iScience.

[123] Liu L., Hu H., Wu J., Koleske A.J., Chen H., Wang N., Yu K., Wu Y., Xiao X., Zhang Q. (2024). Integrin α3 is required for high-frequency repetitive transcranial magnetic stimulation-induced glutamatergic synaptic transmission in mice with ischemia. CNS Neurosci. Ther..

[124] Cherry K.M., Lenze E.J., Lang C.E. (2014). Combining d-cycloserine with motor training does not result in improved general motor learning in neurologically intact people or in people with stroke. J. Neurophysiol..

[125] Lipton S.A. (2006). Paradigm shift in neuroprotection by NMDA receptor blockade: Memantine and beyond. Nat. Rev. Drug Discov..

[126] Rascol O., Fabbri M., Poewe W. (2021). Amantadine in the treatment of Parkinson’s disease and other movement disorders. Lancet Neurol..

[127] Chesnais H., Sloane K.L., Witsch J., Favilla C., Kasner S.E., Rothstein A. (2025). Neurostimulant Use for Rehabilitation and Recovery After Stroke: A Narrative Literature Review. Stroke.

[128] López-Valdés H.E., Clarkson A.N., Ao Y., Charles A.C., Carmichael S.T., Sofroniew M.V., Brennan K.C. (2014). Memantine enhances recovery from stroke. Stroke.

[129] Seyedsaadat S.M., F Kallmes D. (2019). Memantine for the treatment of ischemic stroke: Experimental benefits and clinical lack of studies. Rev. Neurosci..

[130] Liepert J. (2016). Drugs for improvement of motor deficits after stroke. Nervenarzt.

[131] Wu Q.J., Tymianski M. (2018). Targeting NMDA receptors in stroke: New hope in neuroprotection. Mol. Brain.

[132] Thibaut A., Chatelle C., Ziegler E., Bruno M.A., Laureys S., Gosseries O. (2013). Spasticity after stroke: Physiology, assessment and treatment. Brain Inj..

[133] Bethoux F. (2015). Spasticity Management After Stroke. Phys. Med. Rehabil. Clin. N. Am..

[134] Lindsay C., Kouzouna A., Simcox C., Pandyan A.D. (2016). Pharmacological interventions other than botulinum toxin for spasticity after stroke. Cochrane Database Syst. Rev..

[135] Montane E., Brihmat N., Cormier C., Thalamas C., Rousseau V., Tap G., De Boissezon X., Castel-Lacanal E., Marque P. (2025). Effect of Early Treatment of Spasticity After Stroke on Motor Recovery: Protocol for the Baclotox Multicenter, Double-Blind, Double-Dummy Randomized Controlled Trial. JMIR Res. Protoc..

[136] Park K.D., Song M.K. (2024). Intrathecal Baclofen Injection Efficacy for Spasticity Management in Patients With Stroke: A Meta-Analysis. Brain Neurorehabil.

[137] Toda T., Ishida K., Kiyama H., Yamashita T., Lee S. (2014). Down-regulation of KCC2 expression and phosphorylation in motoneurons, and increases the number of in primary afferent projections to motoneurons in mice with post-stroke spasticity. PLoS ONE.

[138] Joy M.T., Carmichael S.T. (2021). Encouraging an excitable brain state: Mechanisms of brain repair in stroke. Nat. Rev. Neurosci..

[139] Murase N., Duque J., Mazzocchio R., Cohen L.G. (2004). Influence of interhemispheric interactions on motor function in chronic stroke. Ann. Neurol..

[140] Napoli A.J., Laderwager S., Zoodsma J.D., Biju B., Mucollari O., Schubel S.K., Aprea C., Sayed A., Morgan K., Napoli A. (2023). Loss of NMDA receptor function during development results in decreased KCC2 expression and increased neurons in the zebrafish forebrain. bioRxiv.

[141] He J.L., Ma L.X., Zhuang Y.X., Wen J.S., Ma L.H., Xiu J.Y., Chen M.Y. (2025). Acupuncture Modulates NMDAR-PP1/Calpain1-KCC2 Pathway to Ameliorate Spinal Hyperexcitability and Spastic Hemiplegia Induced by Ischemic Stroke. J. Integr. Neurosci..

[142] Minematsu K., Fisher M., Li L., Davis M.A., Knapp A.G., Cotter R.E., McBurney R.N., Sotak C.H. (1993). Effects of a novel NMDA antagonist on experimental stroke rapidly and quantitatively assessed by diffusion-weighted MRI. Neurology.

[143] Simon R., Shiraishi K. (1990). N-methyl-D-aspartate antagonist reduces stroke size and regional glucose metabolism. Ann. Neurol..

[144] Bordi F., Pietra C., Ziviani L., Reggiani A. (1997). The glycine antagonist GV150526 protects somatosensory evoked potentials and reduces the infarct area in the MCAo model of focal ischemia in the rat. Exp. Neurol..

[145] Albers G.W., Goldstein L.B., Hall D., Lesko L.M. (2001). Aptiganel hydrochloride in acute ischemic stroke: A randomized controlled trial. JAMA.

[146] Davis S.M., Lees K.R., Albers G.W., Diener H.C., Markabi S., Karlsson G., Norris J. (2000). Selfotel in acute ischemic stroke: Possible neurotoxic effects of an NMDA antagonist. Stroke.

[147] Grotta J., Clark W., Coull B., Pettigrew L.C., Mackay B., Goldstein L.B., Meissner I., Murphy D., LaRue L. (1995). Safety and tolerability of the glutamate antagonist CGS 19755 (Selfotel) in patients with acute ischemic stroke. Results of a phase IIa randomized trial. Stroke.

[148] Lees K.R., Asplund K., Carolei A., Davis S.M., Diener H.C., Kaste M., Orgogozo J.M., Whitehead J., GAIN International Investigators (2000). Glycine antagonist (gavestinel) in neuroprotection (GAIN International) in patients with acute stroke: A randomised controlled trial. Lancet.

[149] Muir K.W., Grosset D.G., Gamzu E., Lees K.R. (1994). Pharmacological effects of the non-competitive NMDA antagonist CNS 1102 in normal volunteers. Br. J. Clin. Pharmacol..

[150] Trotman M., Vermehren P., Gibson C.L., Fern R. (2015). The dichotomy of memantine treatment for ischemic stroke: Dose-dependent protective and detrimental effects. J. Cereb. Blood Flow. Metab..

[151] Cuartero M.I., de la Parra J., Perez-Ruiz A., Bravo-Ferrer I., Duran-Laforet V., Garcia-Culebras A., Garcia-Segura J.M., Dhaliwal J., Frankland P.W., Lizasoain I. (2019). Abolition of aberrant neurogenesis ameliorates cognitive impairment after stroke in mice. J. Clin. Investig..

[152] Tymianski M., Hill M.D., Goyal M., Christenson J., Menon B.K., Swartz R.H., Adams C., Heard K., Kohli Y. (2025). Safety and efficacy of nerinetide in patients with acute ischaemic stroke enrolled in the early window: A post-hoc meta-analysis of individual patient data from three randomised trials. Lancet Neurol..

[153] Ballarin B., Tymianski M. (2018). Discovery and development of NA-1 for the treatment of acute ischemic stroke. Acta Pharmacol. Sin..

[154] Xu Y., Xu L., Xu C., Zhao M., Xu T., Xia L., Wu Y., Cao Y., Han Z. (2024). PSD-95 inhibitor Tat-NR2B9c (NA-1) protects the integrity of the blood-brain barrier after transient middle artery occlusion in rats by downregulating matrix metalloprotease-9 and upregulating endothelial nitric oxide synthase. Brain Res. Bull..

[155] Yang X., Zhao J., Tian H., Nie X., Zheng L., Liu X., Wei Z.Z., Ding Y., Liu L. (2025). Impact of NA-1 on Pericyte-Driven Vasoconstriction and Its Role in No-Reflow During Cerebral Ischemia-Reperfusion. CNS Neurosci. Ther..

[156] Cook D.J., Teves L., Tymianski M. (2012). A translational paradigm for the preclinical evaluation of the stroke neuroprotectant Tat-NR2B9c in gyrencephalic nonhuman primates. Sci. Transl. Med..

[157] Hill M.D., Goyal M., Demchuk A.M., Menon B.K., Field T.S., Guest W.C., Berrouschot J., Bormann A., Pham M., Haeusler K.G. (2025). Efficacy and safety of nerinetide in acute ischaemic stroke in patients undergoing endovascular thrombectomy without previous thrombolysis (ESCAPE-NEXT): A multicentre, double-blind, randomised controlled trial. Lancet.

[158] Hill M.D., Goyal M., Menon B.K., Nogueira R.G., McTaggart R.A., Demchuk A.M., Poppe A.Y., Buck B.H., Field T.S., Dowlatshahi D. (2020). Efficacy and safety of nerinetide for the treatment of acute ischaemic stroke (ESCAPE-NA1): A multicentre, double-blind, randomised controlled trial. Lancet.

[159] Ospel J.M., Goyal M., Menon B.K., Almekhlafi M.A., Zerna C., Nogueira R.G., McTaggart R.A., Demchuk A.M., Poppe A.Y., Rempel J.L. (2025). Factors Influencing Nerinetide Effect on Infarct Volume in Patients Without Alteplase in the Randomized ESCAPE-NA1 Trial. Stroke.

[160] Milani D., Cross J.L., Anderton R.S., Blacker D.J., Knuckey N.W., Meloni B.P. (2017). Neuroprotective efficacy of poly-arginine R18 and NA-1 (TAT-NR2B9c) peptides following transient middle cerebral artery occlusion in the rat. Neurosci. Res..

[161] Meloni B.P., South S.M., Gill D.A., Marriott A.L., Deziel R.A., Jacques A., Blacker D.J., Knuckey N.W. (2019). Poly-Arginine Peptides R18 and R18D Improve Functional Outcomes After Endothelin-1-Induced Stroke in the Sprague Dawley Rat. J. Neuropathol. Exp. Neurol..

[162] Lapchak P.A. (2006). Memantine, an uncompetitive low affinity NMDA open-channel antagonist improves clinical rating scores in a multiple infarct embolic stroke model in rabbits. Brain Res..

[163] Chen B., Wang G., Li W., Liu W., Lin R., Tao J., Jiang M., Chen L., Wang Y. (2017). Memantine attenuates cell apoptosis by suppressing the calpain-caspase-3 pathway in an experimental model of ischemic stroke. Exp. Cell Res..

[164] Montagne A., Hebert M., Jullienne A., Lesept F., Le Behot A., Louessard M., Gauberti M., Orset C., Ali C., Agin V. (2012). Memantine improves safety of thrombolysis for stroke. Stroke.

[165] Jung K.-H., Chu K., Lee S.-T., Park H.-K., Kim J.-H., Kang K.-M., Kim M., Lee S.K., Roh J.-K. (2009). Augmentation of nitrite therapy in cerebral ischemia by NMDA receptor inhibition. Biochem. Biophys. Res. Commun..

[166] Culmsee C., Junker V., Kremers W., Thal S., Plesnila N., Krieglstein J. (2004). Combination Therapy in Ischemic Stroke: Synergistic Neuroprotective Effects of Memantine and Clenbuterol. Stroke.

[167] Kilic U., Yilmaz B., Reiter R.J., Yuksel A., Kilic E. (2013). Effects of memantine and melatonin on signal transduction pathways vascular leakage and brain injury after focal cerebral ischemia in mice. Neuroscience.

[168] Pigretti S.G., Isaac C., Cea C., Esnaola M.M., Riveros M., Tejada Jacob V., Weinberg M., Posadas Martinez M.L., Cirelli D., Burgos M.A. (2023). Pharmacological treatment in early rehabilitation after ischemic stroke. Medicina.

[169] Zhang X., Tian H., Bo H., Zhong L. (2025). NMDAR inhibitor preconditioned mesenchymal stromal cell-derived extracellular vesicles enhance post-stroke recovery by targeting excitotoxicity and neuronal regeneration. Front. Cell Neurosci..

[170] van der Worp H.B., de Haan P., Morrema E., Kalkman C.J. (2005). Methodological quality of animal studies on neuroprotection in focal cerebral ischaemia. J. Neurol..

[171] Davis S.M., Albers G.W., Diener H.C., Lees K.R., Norris J. (1997). Termination of Acute Stroke Studies Involving Selfotel Treatment. ASSIST Steering Committed. Lancet.

[172] Morris G.F., Bullock R., Marshall S.B., Marmarou A., Maas A., Marshall L.F., The Selfotel Investigators (1999). Failure of the competitive N-methyl-D-aspartate antagonist Selfotel (CGS 19755) in the treatment of severe head injury: Results of two phase III clinical trials. J. Neurosurg..

[173] Bullock R. (1995). Strategies for neuroprotection with glutamate antagonists. Extrapolating from evidence taken from the first stroke and head injury studies. Ann. N. Y. Acad. Sci..

[174] Dyker A.G., Edwards K.R., Fayad P.B., Hormes J.T., Lees K.R. (1999). Safety and tolerability study of aptiganel hydrochloride in patients with an acute ischemic stroke. Stroke.

[175] Muir K.W., Lees K.R. (2003). Excitatory amino acid antagonists for acute stroke. Cochrane Database Syst. Rev..

[176] Dyker A.G., Lees K.R. (1999). Safety and tolerability of GV150526 (a glycine site antagonist at the N-methyl-D-aspartate receptor) in patients with acute stroke. Stroke.

[177] Sacco R.L., DeRosa J.T., Haley E.C., Levin B., Ordronneau P., Phillips S.J., Rundek T., Snipes R.G., Thompson J.L. (2001). Glycine antagonist in neuroprotection for patients with acute stroke: GAIN Americas: A randomized controlled trial. JAMA.

[178] Phillips S.J., Dai D., Mitnitski A., Gubitz G.J., Johnston K.C., Koroshetz W.J., Furie K.L., Black S., Heiselman D.E. (2007). Clinical diagnosis of lacunar stroke in the first 6 hours after symptom onset: Analysis of data from the glycine antagonist in neuroprotection (GAIN) Americas trial. Stroke.

[179] Warach S., Kaufman D., Chiu D., Devlin T., Luby M., Rashid A., Clayton L., Kaste M., Lees K.R., Sacco R. (2006). Effect of the Glycine Antagonist Gavestinel on cerebral infarcts in acute stroke patients, a randomized placebo-controlled trial: The GAIN MRI Substudy. Cerebrovasc. Dis..

[180] Haley E.C., Thompson J.L., Levin B., Davis S., Lees K.R., Pittman J.G., DeRosa J.T., Ordronneau P., Brown D.L., Sacco R.L. (2005). Gavestinel does not improve outcome after acute intracerebral hemorrhage: An analysis from the GAIN International and GAIN Americas studies. Stroke.

[181] Christenson J., Hill M.D., Swartz R.H., Adams C., Benavente O., Casaubon L.K., Cheskes S., Ganesh A., Garman J.D., Harris C. (2025). Efficacy and safety of intravenous nerinetide initiated by paramedics in the field for acute cerebral ischaemia within 3 h of symptom onset (FRONTIER): A phase 2, multicentre, randomised, double-blind, placebo-controlled study. Lancet.

[182] Hao J., Mdzinarishvili A., Abbruscato T.J., Klein J., Geldenhuys W.J., Van der Schyf C.J., Bickel U. (2008). Neuroprotection in mice by NGP1-01 after transient focal brain ischemia. Brain Res..

[183] Kafi H., Salamzadeh J., Beladimoghadam N., Sistanizad M., Kouchek M. (2014). Study of the neuroprotective effects of memantine in patients with mild to moderate ischemic stroke. Iran. J. Pharm. Res..

[184] Chen Z.Z., Yang D.D., Zhao Z., Yan H., Ji J., Sun X.L. (2016). Memantine mediates neuroprotection via regulating neurovascular unit in a mouse model of focal cerebral ischemia. Life Sci..

[185] Beladi Moghadam N., Pourheidar E., Ahmadpour F., Kafi H., Salamzadeh J., Nasiri S., Sistanizad M. (2021). The effects of memantine on the serum concentrations of matrix metalloproteinases and neurologic function of patients with ischemic stroke. J. Clin. Neurosci..

[186] Stanton J.A., Williams E.I., Betterton R.D., Davis T.P., Ronaldson P.T. (2022). Targeting organic cation transporters at the blood-brain barrier to treat ischemic stroke in rats. Exp. Neurol..

[187] Xiong Z., Chang L., Qu Y., Pu Y., Wang S., Fujita Y., Ishima T., Chen J., Hashimoto K. (2020). Neuronal brain injury after cerebral ischemic stroke is ameliorated after subsequent administration of (R)-ketamine, but not (S)-ketamine. Pharmacol. Biochem Behav..

[188] Heil L.B.B., Braga C.L., Magalhaes R.F., Antunes M.A., Cruz F.F., Samary C.S., Battaglini D., Robba C., Pelosi P., Silva P.L. (2023). Dexmedetomidine compared to low-dose ketamine better protected not only the brain but also the lungs in acute ischemic stroke. Int. Immunopharmacol..

[189] Gu S.X., Sonkar V.K., Katare P.B., Kumar R., Kruger W.D., Arning E., Bottiglieri T., Lentz S.R., Dayal S. (2020). Memantine Protects From Exacerbation of Ischemic Stroke and Blood Brain Barrier Disruption in Mild But Not Severe Hyperhomocysteinemia. J. Am. Heart Assoc..

[190] Liang Y.B., Guo Y.Q., Song P.P., Zhu Y.H., Zhu P.Z., Liu R.R., Xu J.M., Zhang Y.S. (2020). Memantine ameliorates tau protein deposition and secondary damage in the ipsilateral thalamus and sensory decline following focal cortical infarction in rats. Neurosci. Lett..

[191] Berthier M.L., Green C., Lara J.P., Higueras C., Barbancho M.A., Dávila G., Pulvermüller F. (2009). Memantine and constraint-induced aphasia therapy in chronic poststroke aphasia. Ann. Neurol..

[192] Yu S.P., Gu X., Jiang M.Q., Sastry A., Wu L., Li Y., Wei L. (2025). Combined Preventive and Preconditioning Treatments for the Comorbidity of Alzheimer’s Disease and Ischemic Stroke in a GluN3A Knockout Mouse and a 5xFAD Mouse. Cells.

[193] Abdoulaye I.A., Wu S.S., Chibaatar E., Yu D.F., Le K., Cao X.J., Guo Y.J. (2021). Ketamine Induces Lasting Antidepressant Effects by Modulating the NMDAR/CaMKII-Mediated Synaptic Plasticity of the Hippocampal Dentate Gyrus in Depressive Stroke Model. Neural Plast..

[194] Zhang L.M., Wu Z.Y., Liu J.Z., Li Y., Lv J.M., Wang L.Y., Shan Y.D., Song R.X., Miao H.T., Zhang W. (2023). Subanesthetic dose of S-ketamine improved cognitive dysfunction via the inhibition of hippocampal astrocytosis in a mouse model of post-stroke chronic stress. J. Psychiatr. Res..

[195] Tian J., Xie Y., Ye S., Hu Y., Feng J., Li Y., Lou Z., Ruan L., Wang Z. (2025). S-ketamine ameliorates post-stroke depression in mice via attenuation of neuroinflammation, synaptic restoration, and BDNF pathway activation. Biochem Biophys. Res. Commun..

[196] Milani D., Knuckey N.W., Anderton R.S., Cross J.L., Meloni B.P. (2016). The R18 Polyarginine Peptide Is More Effective Than the TAT-NR2B9c (NA-1) Peptide When Administered 60 Minutes after Permanent Middle Cerebral Artery Occlusion in the Rat. Stroke Res. Treat..

[197] Milani D., Cross J.L., Anderton R.S., Blacker D.J., Knuckey N.W., Meloni B.P. (2017). Delayed 2-h post-stroke administration of R18 and NA-1 (TAT-NR2B9c) peptides after permanent and/or transient middle cerebral artery occlusion in the rat. Brain Res. Bull..

[198] Meythaler J.M., Brunner R.C., Johnson A., Novack T.A. (2002). Amantadine to improve neurorecovery in traumatic brain injury-associated diffuse axonal injury: A pilot double-blind randomized trial. J. Head. Trauma Rehabil..

[199] Barra M.E., Izzy S., Sarro-Schwartz A., Hirschberg R.E., Mazwi N., Edlow B.L. (2020). Stimulant Therapy in Acute Traumatic Brain Injury: Prescribing Patterns and Adverse Event Rates at 2 Level 1 Trauma Centers. J. Intensive Care Med..

[200] Tracy B.M., Silverman M.E., Cordero-Caballero C., Durr E.A., Gelbard R.B. (2021). Dual Neurostimulant Therapy May Optimize Acute Neurorecovery for Severe Traumatic Brain Injuries. J. Surg. Res..

[201] Badre D., Elbeialy M.A.K., Fathy M. (2026). Citicoline-Amantadine Trial in Traumatic Brain Injury: A Prospective Randomized Study. J. Neurotrauma.

[202] Schmitt B., Bauersfeld U., Fanconi S., Wohlrab G., Huisman T.A., Bandtlow C., Baumann P., Superti-Furga A., Martin E., Arbenz U. (1997). The effect of the N-methyl-D-aspartate receptor antagonist dextromethorphan on perioperative brain injury in children undergoing cardiac surgery with cardiopulmonary bypass: Results of a pilot study. Neuropediatrics.

[203] Comi A.M., Highet B.H., Mehta P., Hana Chong T., Johnston M.V., Wilson M.A. (2006). Dextromethorphan protects male but not female mice with brain ischemia. Neuroreport.

[204] Shear D.A., Williams A.J., Sharrow K., Lu X.C., Tortella F.C. (2009). Neuroprotective profile of dextromethorphan in an experimental model of penetrating ballistic-like brain injury. Pharmacol. Biochem Behav..

[205] Posod A., Pinzer K., Urbanek M., Wegleiter K., Keller M., Kiechl-Kohlendorfer U., Griesmaier E. (2014). The common antitussive agent dextromethorphan protects against hyperoxia-induced cell death in established in vivo and in vitro models of neonatal brain injury. Neuroscience.

