# Supplementary material for: NMDA Receptor Mediated Mechanisms in the Post-Stroke Brain: From Physiology to Pathology
Source: Biomolecules. 2026 May 23;16(6):770. doi: 10.3390/biom16060770 (PMC13296956; doi:10.3390/biom16060770)
Supplement: Supplementary file 1 [file biomolecules-16-00770-s001.zip › Supplementary Table S1.pdf]

Study (Author, Phase/Type)	Drug Name	Intervention Timing	Dose & Toxicity	Preclinical vs. Clinical Outcome	Mechanistic Insights & Translational Gap
Class I: Competitive NMDA Receptor Antagonists					
Simon et al.[1] (Preclinical)	Selfotel (CGS 19755)	Preclinical: 5 min pre-/post-ischemia	10 mg/kg i.v.; Toxicity: NR	Preclinical: Infarct volume reduced (~14.7% → ~6.5%); attenuated hypermetabolism	Efficacy dependent on ultra-early intervention
van der Worp et al.[2] (Review)	Selfotel	Preclinical: Median 10 min post-ischemia	NR	Preclinical: Neuroprotection observed	Overestimation due to methodological limitations and non-clinically relevant timing
Grotta et al.[3] (Phase IIa)	Selfotel	Clinical: ≤12 h post-onset	1.0–2.0 mg/kg i.v. (MTD: 1.5 mg/kg); Dose-limiting toxicity: Severe neuropsychiatric effects (agitation, hallucinations, confusion, delirium)	Clinical: No efficacy	Inability to achieve neuroprotective concentrations due to dose-limiting CNS toxicity
Davis et al.[4,5](Phase III ASSIST)	Selfotel	Clinical: ≤6 h post-onset	1.5 mg/kg i.v.; Toxicity: Increased early (30-day) mortality	Clinical: Early termination (safety imbalance)	Signal of potential neurotoxicity in ischemic human brain
Morris et al. [6] (Phase III TBI)	Selfotel	Clinical: Post-resuscitation	MTD: 1.5 mg/kg; Toxicity: NR	Clinical: No efficacy; early termination after safety concerns in concurrent stroke trials	Dose restriction and clinical heterogeneity limiting efficacy
Bullock[7] (Review)	Selfotel	Preclinical: 5–30 min post-injury; Clinical: 6–24 h post-onset	Stroke MTD: 1.5 mg/kg; TBI effective dose: >3 mg/kg	Preclinical: Neuroprotection; Clinical: No efficacy	Dose-ceiling effect due to psychotoxicity preventing efficacious dosing
Class II: Non-competitive NMDA Receptor Antagonists					
Minematsu et al.[8] (Preclinical)	Aptiganel (Cerestat)	Preclinical: 15 min post-MCAO	1.12 mg/kg i.v. bolus, then 0.33 mg/kg/h for 3 h; Toxicity: NR	Preclinical: Infarct volume reduced (~66%)	Hyperacute neuroprotection demonstrated
Muir et al. [9] (Phase I)	Aptiganel	Clinical: Healthy	15-min i.v. infusion (MTD: 30	Clinical: Dose escalation halted	Rapid CNS penetration

Study (Author, Phase/Type)	Drug Name	Intervention Timing	Dose & Toxicity	Preclinical vs. Clinical Outcome	Mechanistic Insights & Translational Gap
		volunteers	μg/kg); Toxicity: Sedation, increased BP/pulse, CNS excitation	(adverse effects)	associated with acute adverse effects
Dyker et al.[10] (Phase II)	Aptiganel	Clinical: ≤24 h post-onset	4.5 mg i.v. bolus, then 0.75 mg/h for 12 h; Dose-limiting toxicity: Increased systolic BP (~30 mmHg), CNS excitation	Clinical: Neuroprotective plasma concentrations achieved	Therapeutic window limited by cardiovascular and CNS toxicity
Albers et al.[11](Phase II/III)	Aptiganel	Clinical: ≤6 h post-onset	5 mg i.v. bolus, then 0.75 mg/h for 12 h; Toxicity: Increased 120-day mortality	Clinical: No efficacy; slower neurological recovery	Possible detrimental effects of glutamate blockade; delayed administration
Muir & Lees[12](Meta-analysis)	Aptiganel	Clinical: ≤24 h post-onset	Toxicity: Psychiatric adverse events	Clinical: Trend toward increased mortality and worse outcomes	Lack of clinical benefit despite preclinical efficacy
Class III: Selective Glycine-Site NMDA Receptor Antagonists					
Bordi et al.[13](Preclinical)	Gavestinel (GV150526)	Preclinical: ≤6 h post-MCAO	3 mg/kg i.v.; Toxicity: NR	Preclinical: Reduced infarct volume; preserved cortical responses	Sustained neuroprotection within extended time window
Dyker & Lees[14](Phase II)	Gavestinel	Clinical: 6–12 h post-onset	≤800 mg loading dose, then 200 mg q12h; Toxicity: No significant hemodynamic or CNS events	Clinical: High tolerability	Improved safety profile compared with earlier NMDA antagonists
Sacco et al.[15](Phase III GAIN Americas)	Gavestinel	Clinical: ≤6 h post-onset	800 mg i.v. loading, then 200 mg q12h (5 doses); Toxicity: No significant toxicity observed	Clinical: No improvement in functional outcomes	Single target intervention may be insufficient for clinical benefit
Phillips et al.[16] (Phase III Subgroup)	Gavestinel	Clinical: ≤6 h post-onset	800 mg i.v. loading, then 200 mg q12h; Toxicity: NR	Clinical: No benefit in lacunar stroke subgroup	Drug cannot intervene in underlying vascular pathological processes

Study (Author, Phase/Type)	Drug Name	Intervention Timing	Dose & Toxicity	Preclinical vs. Clinical Outcome	Mechanistic Insights & Translational Gap
Warach et al. [17](Phase III MRI Substudy)	Gavestinel	Clinical: ≤6 h post-onset	800 mg i.v. bolus, then 200 mg q12h (5 doses); Toxicity: NR	Clinical: No difference in infarct volume change; failed to reduce infarct even in DWI-PWI mismatch subgroup	Target saturation: doses achieving receptor saturation failed to arrest infarct progression; 6-hour window may be too late
Haley et al.[18] (Phase III ICH Analysis)	Gavestinel	Clinical: ≤6 h post-onset	800 mg loading dose, then 200 mg q12h (5 doses); Toxicity: Serious adverse events similar to placebo	Clinical: No substantial benefit in patients with primary intracerebral hemorrhage	Consistent with neutral results previously observed in ischemic stroke
Mechanistic Overview					
Ikonomidou & Turski[19](Review)	NMDA antagonists (class effect)	Preclinical: Antecedent or 10–30 min; Clinical: Typically ~6 h	Not applicable	Preclinical: Effective with immediate intervention; Clinical: Failed across trials	Missed excitotoxic window and interference with physiological synaptic survival signaling
Class IV: PSD-95–Disrupting Peptides					
Ballarin and Tymianski [20] (Review)	Nerinetide (NA-1 / Tat-NR2B9c)	Not applicable	Not applicable	Preclinical: Identified PSD-95/GluN2B interaction as a key excitotoxic culprit; NA-1 showed potent protection across multiple species	Uncouples PSD-95 from neurotoxic signaling pathways without directly antagonizing the NMDA receptor
Cook, et al. [21] (Preclinical)	Nerinetide	Preclinical: 1 h post-emboli	2.6 mg/kg i.v. (10-min infusion); Toxicity: No significant adverse physiological or neurological effects observed	Preclinical: Infarct volume reduced by 56% (DWI) and 60% (T2); number of strokes reduced by 64%	Validated efficacy in gyrencephalic primates mimicking clinical settings; critical 1-hour hyper-acute therapeutic window
Hill, et al.[22] (Phase III)	Nerinetide	Clinical: ⩽12 h post-onset (during/after EVT)	2.6 mg/kg i.v. (single dose); Toxicity: Serious adverse events similar to placebo (12.2% vs. 12.0%)	Clinical: No significant difference in primary outcome (mRS 0–2 at 90 days) in the overall population (59.3% vs. 59.2%)	Alteplase interaction: plasmin-induced proteolytic cleavage degrades the active peptide during concurrent thrombolysis

Study (Author, Phase/Type)	Drug Name	Intervention Timing	Dose & Toxicity	Preclinical vs. Clinical Outcome	Mechanistic Insights & Translational Gap
Hill, et al. [23] (Phase III ESCAPE-NEXT)	Nerinetide	Clinical: ≤12 h post-onset (undergoing EVT without previous thrombolysis)	2.6 mg/kg i.v. (single dose); Toxicity: Serious adverse events occurred equally between groups, no safety concerns	Clinical: No significant difference in mRS 0–2 at 90 days (45% vs. 46%) or median infarct volumes	Short window for neuroprotection to have a benefit before rapid reperfusion; difficult to benefit slow progressors in late windows
Christenson, et al. [24] (Phase II FRONTIER)	Nerinetide	Clinical: ≤3 h post-onset (prehospital paramedic administration)	2.6 mg/kg i.v. (single dose); Toxicity: No safety concerns (well tolerated across suspected stroke subtypes)	Clinical: Overall suspected stroke cohort neutral; improved functional outcomes in ischaemic stroke subgroup receiving reperfusion	Prehospital administration avoids alteplase-induced cleavage; ultra-early intervention (<3h) prevents cell death prior to clot dissolution
Tymianski, et al. [25] (Meta-analysis)	Nerinetide	Clinical: ≤3 h post-onset (selected for reperfusion without previous thrombolysis)	2.6 mg/kg i.v.; Toxicity: No safety concerns (mortality 16% vs. 18%)	Clinical: Significant improvement in 90-day favorable outcome (56% vs. 48%); reduced stroke worsening and infarct volumes	Benefit restored by matching preclinical criteria; temporising effect maximized with prolonged interval to reperfusion (dwell time)
Ospel, et al.[26] (Secondary imaging analysis)	Nerinetide	Clinical: ≤12 h post-onset (subgroup without alteplase)	2.6 mg/kg i.v. (single dose); Toxicity: Small, transient, nonsignificant increase in hypotensive events	Clinical: Strongly associated with smaller final infarct volumes (median 26.7 mL vs. 39.2 mL)	Structural neuroprotection without alteplase; attenuates harm from high blood pressure, anesthesia, and incomplete reperfusion

Table S1: Clinical trial outcomes of classic NMDA receptor antagonist drugs for ischemic stroke treatment

Abbreviations: BP: blood pressure; CNS: central nervous system; ICH: intracerebral hemorrhage; i.v.: intravenous; MCAO: middle cerebral artery occlusion; MTD: maximum tolerated dose; NR: not reported; q12h: every 12 hours; TBI: traumatic brain injury.

Note: Unless otherwise specified, preclinical efficacy data are derived from rodent permanent MCAO models.

1. Simon, R.; Shiraishi, K. N-methyl-D-aspartate antagonist reduces stroke size and regional glucose metabolism. *Ann. Neurol.* **1990**, *27*, 606-611, doi:10.1002/ana.410270604.
2. van der Worp, H.B.; de Haan, P.; Morrema, E.; Kalkman, C.J. Methodological quality of animal studies on neuroprotection in focal cerebral ischaemia. *J. Neurol.* **2005**, *252*, 1108-1114, doi:10.1007/s00415-005-0802-3.

3. Grotta, J.; Clark, W.; Coull, B.; Pettigrew, L.C.; Mackay, B.; Goldstein, L.B.; Meissner, I.; Murphy, D.; LaRue, L. Safety and tolerability of the glutamate antagonist CGS 19755 (Selfotel) in patients with acute ischemic stroke. Results of a phase IIa randomized trial. *Stroke* **1995**, *26*, 602-605, doi:10.1161/01.str.26.4.602.
4. Davis, S.M.; Lees, K.R.; Albers, G.W.; Diener, H.C.; Markabi, S.; Karlsson, G.; Norris, J. Selfotel in acute ischemic stroke : possible neurotoxic effects of an NMDA antagonist. *Stroke* **2000**, *31*, 347-354, doi:10.1161/01.str.31.2.347.
5. Davis, S.M.; Albers, G.W.; Diener, H.C.; Lees, K.R.; Norris, J. Termination of Acute Stroke Studies Involving Selfotel Treatment. ASSIST Steering Committed. *Lancet* **1997**, *349*, 32, doi:10.1016/s0140-6736(05)62166-6.
6. Morris, G.F.; Bullock, R.; Marshall, S.B.; Marmarou, A.; Maas, A.; Marshall, L.F. Failure of the competitive N-methyl-D-aspartate antagonist Selfotel (CGS 19755) in the treatment of severe head injury: results of two phase III clinical trials. The Selfotel Investigators. *J. Neurosurg.* **1999**, *91*, 737-743, doi:10.3171/jns.1999.91.5.0737.
7. Bullock, R. Strategies for neuroprotection with glutamate antagonists. Extrapolating from evidence taken from the first stroke and head injury studies. *Ann. N. Y. Acad. Sci.* **1995**, *765*, 272-278; discussion 298, doi:10.1111/j.1749-6632.1995.tb16584.x.
8. Minematsu, K.; Fisher, M.; Li, L.; Davis, M.A.; Knapp, A.G.; Cotter, R.E.; McBurney, R.N.; Sotak, C.H. Effects of a novel NMDA antagonist on experimental stroke rapidly and quantitatively assessed by diffusion-weighted MRI. *Neurology* **1993**, *43*, 397-397, doi:10.1212/WNL.43.2.397.
9. Muir, K.W.; Grosset, D.G.; Gamzu, E.; Lees, K.R. Pharmacological effects of the non-competitive NMDA antagonist CNS 1102 in normal volunteers. *Br. J. Clin. Pharmacol.* **1994**, *38*, 33-38, doi:10.1111/j.1365-2125.1994.tb04318.x.
10. Dyker, A.G.; Edwards, K.R.; Fayad, P.B.; Hormes, J.T.; Lees, K.R. Safety and tolerability study of aptiganel hydrochloride in patients with an acute ischemic stroke. *Stroke* **1999**, *30*, 2038-2042, doi:10.1161/01.str.30.10.2038.
11. Albers, G.W.; Goldstein, L.B.; Hall, D.; Lesko, L.M. Aptiganel hydrochloride in acute ischemic stroke: a randomized controlled trial. *JAMA* **2001**, *286*, 2673-2682, doi:10.1001/jama.286.21.2673.
12. Muir, K.W.; Lees, K.R. Excitatory amino acid antagonists for acute stroke. *Cochrane Database Syst. Rev.* **2003**, *2003*, Cd001244, doi:10.1002/14651858.Cd001244.
13. Bordi, F.; Pietra, C.; Ziviani, L.; Reggiani, A. The glycine antagonist GV150526 protects somatosensory evoked potentials and reduces the infarct area in the MCAo model of focal ischemia in the rat. *Exp. Neurol.* **1997**, *145*, 425-433, doi:10.1006/exnr.1997.6442.
14. Dyker, A.G.; Lees, K.R. Safety and tolerability of GV150526 (a glycine site antagonist at the N-methyl-D-aspartate receptor) in patients with acute stroke. *Stroke* **1999**, *30*, 986-992, doi:10.1161/01.str.30.5.986.
15. Sacco, R.L.; DeRosa, J.T.; Haley, E.C., Jr.; Levin, B.; Ordronneau, P.; Phillips, S.J.; Rundek, T.; Snipes, R.G.; Thompson, J.L. Glycine antagonist in neuroprotection for patients with acute stroke: GAIN Americas: a randomized controlled trial. *JAMA* **2001**, *285*, 1719-1728, doi:10.1001/jama.285.13.1719.

16. Phillips, S.J.; Dai, D.; Mitnitski, A.; Gubitz, G.J.; Johnston, K.C.; Koroshetz, W.J.; Furie, K.L.; Black, S.; Heiselman, D.E. Clinical diagnosis of lacunar stroke in the first 6 hours after symptom onset: analysis of data from the glycine antagonist in neuroprotection (GAIN) Americas trial. *Stroke* **2007**, *38*, 2706-2711, doi:10.1161/strokeaha.107.487744.
17. Warach, S.; Kaufman, D.; Chiu, D.; Devlin, T.; Luby, M.; Rashid, A.; Clayton, L.; Kaste, M.; Lees, K.R.; Sacco, R.; et al. Effect of the Glycine Antagonist Gavestinel on cerebral infarcts in acute stroke patients, a randomized placebo-controlled trial: The GAIN MRI Substudy. *Cerebrovasc. Dis.* **2006**, *21*, 106-111, doi:10.1159/000090208.
18. Haley, E.C., Jr.; Thompson, J.L.; Levin, B.; Davis, S.; Lees, K.R.; Pittman, J.G.; DeRosa, J.T.; Ordronneau, P.; Brown, D.L.; Sacco, R.L. Gavestinel does not improve outcome after acute intracerebral hemorrhage: an analysis from the GAIN International and GAIN Americas studies. *Stroke* **2005**, *36*, 1006-1010, doi:10.1161/01.STR.0000163053.77982.8d.
19. Ikonomidou, C.; Turski, L. Why did NMDA receptor antagonists fail clinical trials for stroke and traumatic brain injury? *Lancet Neurol* **2002**, *1*, 383-386, doi:10.1016/s1474-4422(02)00164-3.
20. Ballarin, B.; Tymianski, M. Discovery and development of NA-1 for the treatment of acute ischemic stroke. *Acta Pharmacol. Sin.* **2018**, *39*, 661-668, doi:10.1038/aps.2018.5.
21. Cook, D.J.; Teves, L.; Tymianski, M. A translational paradigm for the preclinical evaluation of the stroke neuroprotectant Tat-NR2B9c in gyrencephalic nonhuman primates. *Sci. Transl. Med.* **2012**, *4*, 154ra133, doi:10.1126/scitranslmed.3003824.
22. Hill, M.D.; Goyal, M.; Menon, B.K.; Nogueira, R.G.; McTaggart, R.A.; Demchuk, A.M.; Poppe, A.Y.; Buck, B.H.; Field, T.S.; Dowlatshahi, D.; et al. Efficacy and safety of nerinetide for the treatment of acute ischaemic stroke (ESCAPE-NA1): a multicentre, double-blind, randomised controlled trial. *Lancet* **2020**, *395*, 878-887, doi:10.1016/s0140-6736(20)30258-0.
23. Hill, M.D.; Goyal, M.; Demchuk, A.M.; Menon, B.K.; Field, T.S.; Guest, W.C.; Berrouschot, J.; Bormann, A.; Pham, M.; Haeusler, K.G.; et al. Efficacy and safety of nerinetide in acute ischaemic stroke in patients undergoing endovascular thrombectomy without previous thrombolysis (ESCAPE-NEXT): a multicentre, double-blind, randomised controlled trial. *Lancet* **2025**, *405*, 560-570, doi:10.1016/s0140-6736(25)00194-1.
24. Christenson, J.; Hill, M.D.; Swartz, R.H.; Adams, C.; Benavente, O.; Casaubon, L.K.; Cheskes, S.; Ganesh, A.; Garman, J.D.; Harris, C.; et al. Efficacy and safety of intravenous nerinetide initiated by paramedics in the field for acute cerebral ischaemia within 3 h of symptom onset (FRONTIER): a phase 2, multicentre, randomised, double-blind, placebo-controlled study. *Lancet* **2025**, *405*, 571-582, doi:10.1016/s0140-6736(25)00193-x.

25. Tymianski, M.; Hill, M.D.; Goyal, M.; Christenson, J.; Menon, B.K.; Swartz, R.H.; Adams, C.; Heard, K.; Kohli, Y. Safety and efficacy of nerinetide in patients with acute ischaemic stroke enrolled in the early window: a post-hoc meta-analysis of individual patient data from three randomised trials. *Lancet Neurol.* **2025**, *24*, 208-217, doi:10.1016/s1474-4422(24)00515-5.
26. Ospel, J.M.; Goyal, M.; Menon, B.K.; Almekhlafi, M.A.; Zerna, C.; Nogueira, R.G.; McTaggart, R.A.; Demchuk, A.M.; Poppe, A.Y.; Rempel, J.L.; et al. Factors Influencing Nerinetide Effect on Infarct Volume in Patients Without Alteplase in the Randomized ESCAPE-NA1 Trial. *Stroke* **2025**, *56*, 14-21, doi:10.1161/strokeaha.124.048601.
