# Supplementary material for: NMDA Receptor Mediated Mechanisms in the Post-Stroke Brain: From Physiology to Pathology
Source: Biomolecules. 2026 May 23;16(6):770. doi: 10.3390/biom16060770 (PMC13296956; doi:10.3390/biom16060770)
Supplement: Supplementary file 1 [file biomolecules-16-00770-s001.zip › Supplementary Table S2.pdf]

Reference	Intervention	NMDAR Subtype / Mechanistic Axis	Treatment Paradigm	Quantitative Outcomes	Mechanistic Interpretation & Translational Relevance
Block 1: Acute Excitotoxicity and Early Neuroprotection in Ischemic Stroke					
Culmsee et al.[1]	Memantine + Clenbuterol	Uncompetitive NMDAR blockade; β2-AR agonism	20 + 0.3–3 mg/kg (i.p.); ≤30 min post-ischemia	↓ infarct size vs monotherapy (−29%); window 5→30 min	Combination therapy extends the therapeutic window compared with monotherapy
Lapchak et al. [2]	Memantine	Uncompetitive NMDAR open-channel blockade	25 mg/kg (i.v. infusion); 5–60 min post-embolization	↑ behavioral scores; ↑ P50 (+189%)	Demonstrates efficacy in an embolic stroke model and supports intravenous administration
Hao et al.[3]	Memantine / NGP1-01	Uncompetitive NMDAR blockade; VGCC inhibition	20 mg/kg (i.p.); 15 min, 24 h, 48 h post-reperfusion	↓ infarct volume (−25–30%); ↑ motor function	Demonstrates sustained subacute-phase neuroprotection
Jung et al. [4]	Memantine + Sodium nitrite	Uncompetitive NMDAR open-channel blockade	20 mg/kg (i.p.); 1.5–4.5 h post-ischemia	↓ infarct volume vs nitrite alone (−42%); window extended to 4.5 h	Reduces nitrite-associated toxicity and extends the therapeutic window
Montagne et al.[5]	Memantine	Extrasynaptic NMDAR blockade	20 mg/kg (i.v. bolus); pre-rtPA	↓ lesion volume; reduced rtPA-associated toxicity	Reduces lesion volume and rtPA-associated toxicity, supporting extension of the thrombolysis window
Fallah A, et al. [6]	Memantine	NMDAR blockade	60 mg/day (p.o.); ≤24 h post-stroke for 5 days	Assessed safety and clinical scores	Explores high-dose add-on therapy in acute ischemic stroke
Kilic et al.[7]	Memantine + Melatonin	NMDAR blockade; antioxidant	20 + 4 mg/kg (i.p.); 15 min post-ischemia	↓ infarct volume vs monotherapy (−41.2% vs −18.2%)	Demonstrates enhanced neuroprotection with combination therapy

Reference	Intervention	NMDAR Subtype / Mechanistic Axis	Treatment Paradigm	Quantitative Outcomes	Mechanistic Interpretation & Translational Relevance
Trotman et al.[8]	Memantine	Uncompetitive NMDAR open-channel blockade	0.02–20 mg/kg/day (s.c. infusion); prophylactic–48 h	Low dose: ↓ infarct (−30–50%); high dose: ↑ injury	Demonstrates dose-dependent bidirectional effects, with low doses reducing injury and high doses increasing injury
Chen et al.[9]	Memantine	NMDAR blockade	20 mg/kg (loading dose); immediately post-MCAO	↓ Evans Blue dye extravasation	Reduces BBB permeability following ischemia
Chen et al.[10]	Memantine	Extrasynaptic NMDAR blockade	20 mg/kg (i.p.) + 1 mg/kg/12 h; immediately post-MCAO	↓ infarct volume (33.2% → 21.9%)	Reduces infarct volume through extrasynaptic NMDAR inhibition
Beladi Moghadam et al.[11]	Memantine	NMDAR blockade	60 mg/day → 20 mg/day (p.o.); ≤24 h post-stroke	Assessed acute-to-maintenance efficacy	Evaluates a high-dose to maintenance dosing regimen in acute ischemic stroke
Stanton et al.[12]	Memantine	NMDAR blockade; Oct1/Oct2 transport dependent	5 mg/kg (i.v.); 2 h post-reperfusion	↓ infarct volume; effect abolished by cimetidine co-administration	Demonstrates that neuroprotective efficacy depends on BBB Oct1/Oct2 transport
Xiong et al. [13]	(R)-ketamine	Uncompetitive NMDAR open-channel blockade	10 mg/kg (i.p.); 1–24 h post-ischemia	↓ infarct volume vs (S)-enantiomer	Demonstrates greater neuroprotection of (R)-ketamine compared with (S)-ketamine
Heil et al.[14]	Dexmedetomidine vs S-ketamine	NMDAR blockade vs α2-AR agonism	0.5–1.5 mg/kg/h (i.v. infusion); 24 h post-ischemia	DEX: ↓ TNF-α; alleviated brain/lung inflammation	Shows that dexmedetomidine reduces inflammatory markers compared with S-ketamine
Block 2: Post-Stroke Recovery, Plasticity, and Comorbidity Modulation					

Reference	Intervention	NMDAR Subtype / Mechanistic Axis	Treatment Paradigm	Quantitative Outcomes	Mechanistic Interpretation & Translational Relevance
Cuartero et al.[15]	Memantine	Uncompetitive NMDAR open-channel blockade	25 mg/kg (i.p., weekly); 7 days post-infarction for 4 weeks	↑ hippocampal neurogenesis; ↑ long-term memory impairment	Aberrant neurogenesis is associated with cognitive impairment; its suppression improves post-stroke outcomes
Gu et al.[16]	Memantine	NMDAR blockade	30 mg/kg/day (p.o.); 14 days pre-stroke	Effective in mild hyperhomocysteinemia; ineffective in severe condition	Identifies pathological severity limits of prophylactic neuroprotection
Liang et al.[17]	Memantine	NMDAR blockade	20 mg/kg (i.p.); 24 h post-ischemia for 7 days	↑ intact VPN neurons; ↓ reactive astrogliosis	Protects secondary thalamic damage without altering primary cortical infarct
Berthier et al.[18]	Memantine + CIAT	NMDAR blockade	20 mg/day (p.o.); chronic post-stroke aphasia	↑ aphasia quotient (AQ); ↑ naming and language recovery	Demonstrates clinical efficacy of NMDAR blockade in enhancing experience-dependent cortical plasticity
Yu et al.[19]	Memantine	Extrasynaptic NMDAR blockade	10 mg/kg/day (p.o.); 4–8 weeks pre-stroke	↓ infarct volume; ↓ pro-apoptotic proteins	Provides long-term preventive efficacy against chronic excitotoxicity
Yu et al.[20]	Memantine	Extrasynaptic NMDAR blockade; pharmacological preconditioning	10 mg/kg/day (p.o.); 4 weeks pre-stroke	↓ infarct volume; ↑ ischemic tolerance in 5xFAD/GluN3A KO	Enhances ischemic tolerance in Alzheimer's disease comorbidity models
Zhang et al. [21]	M-EVs (Memantine-preconditioned)	NMDAR preconditioning	50 μg (i.v.); 24 h post-ischemia	↑ motor and spatial memory recovery vs conventional EVs	Enhances functional recovery compared with conventional EV treatment
Abdoulaye et al. [22]	Ketamine	NMDAR blockade; PSD-95 modulation	25 μg (stereotaxic injection); chronic phase (post-CUMS)	Reversed depressive-like behaviors; restored PSD95 binding	Reverses depressive-like behaviors and restores PSD95 binding

Reference	Intervention	NMDAR Subtype / Mechanistic Axis	Treatment Paradigm	Quantitative Outcomes	Mechanistic Interpretation & Translational Relevance
Zhang et al. [23]	S-ketamine	NMDAR blockade	15 mg/kg (i.p.); 4 weeks post-stroke	Reversed spatial memory impairment; ↓ anxiety/depression	Reports cognitive and emotional benefits in chronic post-stroke stress
Tian et al. [24]	S-ketamine	Uncompetitive NMDAR open-channel blockade	10 mg/kg (i.p.); chronic phase	Alleviated depressive-like behaviors; ↑ dendritic spine density	Increases dendritic spine density and alleviates depressive-like behaviors
Block 3: Translational Perspectives in Neuroprotection					
Milani et al.[25]	NA-1 (TAT-NR2B9c) vs. Poly-arginine R18	R18: Pleiotropic/multi-target mechanisms (attenuates excitotoxicity, calcium influx, and preserves mitochondrial function);NA-1: Uncouples NMDARs from PSD-95 protein	30–1000 nmol/kg (i.v.); 60 min post-MCAO (90 min transient) in rats	NA-1 (1000 nmol/kg): ↓ infarct volume (26.1%); R18: ↓ infarct volume (35.1%), ↓ swelling (46.1%)	In severe stroke without reperfusion (mimicking failed clinical recanalization), R18's broad cell-penetrating protective mechanisms demonstrate superior efficacy compared to singular PSD-95 blockade.
Milani et al.[26]	NA-1 (TAT-NR2B9c) vs. Poly-arginine R18	R18: Pleiotropic/multi-target mechanisms (attenuates excitotoxicity, calcium influx, and preserves mitochondrial function);NA-1: Uncouples NMDARs from PSD-95 protein	100–1000 nmol/kg (i.v.); 60 min post-permanent MCAO in rats	R18 (1000 nmol/kg): ↓ total infarct volume (24%, P=0.013); NA-1: ↓ infarct volume (7%, non-significant)	R18 have superior efficacy and wider therapeutic window compared to NA-1 in a severe permanent ischemia model.
Milani et al.[27]	NA-1 (TAT-NR2B9c) vs. Poly-arginine R18	R18: Pleiotropic/multi-target mechanisms (attenuates excitotoxicity, calcium influx, and preserves mitochondrial function);NA-1: Uncouples	100–1000 nmol/kg (i.v. or i.a.); delayed administration 2 h post-MCAO (permanent or 3-h	NA-1: No significant reduction in infarct volume or functional improvement at 2 h post-stroke. R18: Did not reduce infarct volume,	Explicitly defines a strict therapeutic time window (<2 hours) for infarct volume reduction, providing direct preclinical evidence for

Reference	Intervention	NMDAR Subtype / Mechanistic Axis	Treatment Paradigm	Quantitative Outcomes	Mechanistic Interpretation & Translational Relevance
		NMDARs from PSD-95 protein	transient) in rats	but ↓ cerebral edema (p=0.006) and ↑ functional outcome (tape removal, p=0.04).	translational failures associated with delayed administration.
Xu et al.[28]	Tat-NR2B9c (NA-1)	PSD-95 inhibition; BBB protection via MMP-9 downregulation and eNOS upregulation	7.5 mg/kg (i.v.); 1 h post-tMCAO in rats	↓ cerebral infarction and edema; mitigated blood-brain barrier injury; ↑ tight junction proteins	Expands the neuroprotective mechanism of NA-1 to the neurovascular unit, demonstrating it preserves blood-brain barrier integrity.
Yang et al.[29]	Tat-NR2B9c (NA-1)	PSD-95 inhibition; Mitigation of pericyte-driven vasoconstriction; Reduction of no-reflow phenomenon	Preclinical: Tested in rat MCAO/R models and in vitro pericyte cultures	↓ no-reflow area; ↑ microvascular patency; improved cerebral blood flow after recanalization	Demonstrates that NA-1 preserves microvascular perfusion by preventing pericyte contraction, addressing the "no-reflow" challenge in stroke therapy.
Cook et al.[30]	Tat-NR2B9c (NA-1)	PSD-95/NMDAR uncoupling	1000 nmol/kg (i.v.); post-ischemia (MCAO) in non-human primates (macaques)	↓ infarct volume; preserved neurological and motor function	Validated the profound neuroprotective efficacy of PSD-95 inhibition in a gyrencephalic brain highly homologous to humans, successfully bridging the gap between rodent models and clinical trials.
Meloni et al.[31]	Poly-arginine R18 and R18D	Arginine-mediated neuroprotection / Sustained neurobehavioral rescue	100–1000 nmol/kg (i.v.); 60 min post Endothelin-1-induced MCAO in rats	Improved functional recovery sustained up to 56 days post-stroke	Extends the preclinical evidence for R18 beyond acute infarct volume reduction, demonstrating long-term behavioral and motor function recovery in a highly

Reference	Intervention	NMDAR Subtype / Mechanistic Axis	Treatment Paradigm	Quantitative Outcomes	Mechanistic Interpretation & Translational Relevance
					relevant stroke model.
Block 4: Non-Stroke CNS Injury Models (TBI & Others)					
Meythaler et al. [32]	Amantadine	Low-affinity NMDAR blockade; DA agonism	200 mg/day (p.o.); 4 days–6 weeks post-injury	↑ daytime alertness; ↑ recovery trajectory	Improves recovery trajectory and daytime alertness in subacute TBI
Barra et al. [33]	Amantadine + Modafinil / Methylphenidate	NMDAR blockade; DA/NE modulation	100–200 mg/day (Amantadine); median 11 days post-injury	Assessed prescribing patterns and adverse events	Explores acute-phase stimulant therapy feasibility in severe TBI
Tracy et al.[34]	Amantadine + Methylphenidate	NMDAR blockade; DA/NE reuptake inhibition	100 mg + 10 mg; acute hospitalization phase	Evaluated acute neurorecovery endpoints	Investigates early dual neurostimulant efficacy in TBI
Badre et al. [35]	Amantadine + Citicoline	NMDAR blockade; phosphatidylcholine precursor	100 mg (Amantadine); acute hospitalization phase	Assessed functional outcomes	Evaluates synergistic potential of combined neurostimulants
Schmitt et al. [36]	Dextromethorphan	NMDAR blockade	36–38 mg/kg/day (i.v.); 24 h pre-op–48 h post-op	Assessed perioperative neuroprotection	Investigates neuroprotective feasibility in pediatric cardiac surgery
Comi et al. [37]	Dextromethorphan	NMDAR blockade	30 mg/kg (s.c.); immediately post-ischemia	Male: ↓ injury scores (13.0 → 7.5); female: no effect	Reports sex-dependent neuroprotection in developmental ischemia
Shear et al. [38]	Dextromethorphan	NMDAR blockade; Sigma-1 agonism	1.0–5.0 mg/kg (i.v. bolus); 30 min–48 h post-injury	↓ infarct volume; improved motor/cognitive function	Demonstrates anatomical and functional protection in penetrating TBI
Posod et al.[39]	Dextromethorphan	NMDAR blockade; Sigma-1 agonism	5–25 mg/kg (i.p.); immediately	↓ hyperoxia-induced apoptosis; ↓ caspase-3	Identifies prophylactic cytoprotection in neonatal white

Reference	Intervention	NMDAR Subtype / Mechanistic Axis	Treatment Paradigm	Quantitative Outcomes	Mechanistic Interpretation & Translational Relevance
			pre-hyperoxia		and gray matter

Table S2. Mechanistic exploration and expanded application of novel NMDA-related neuroprotective therapies

Abbreviations: AD, Alzheimer's disease; BBB, blood-brain barrier; BID, twice a day; CUMS, chronic unpredictable mild stress; CYP2D6, cytochrome P450 2D6; DEX, dexmedetomidine; EVs, extracellular vesicles; i.p., intraperitoneal; i.v., intravenous; MCAO, middle cerebral artery occlusion; NMDA, N-methyl-D-aspartate; NMDAR, NMDA receptor; Oct1/Oct2, organic cation transporters 1 and 2; p.o., per os (oral administration); P50, clot weight producing neurological dysfunction in 50% of animals; rtPA, recombinant tissue plasminogen activator; s.c., subcutaneous; TBI, traumatic brain injury; TNF-α, tumor necrosis factor alpha; VGCC, voltage-gated calcium channel; VPN, ventroposterior thalamic nucleus; β2-AR, β2-adrenergic receptor; NO, nitric oxide.

1. Culmsee, C.; Junker, V.; Kremers, W.; Thal, S.; Plesnila, N.; Krieglstein, J. Combination therapy in ischemic stroke: synergistic neuroprotective effects of memantine and clenbuterol. *Stroke* **2004**, *35*, 1197-1202, doi:10.1161/01.STR.0000125855.17686.6d.
2. Lapchak, P.A. Memantine, an uncompetitive low affinity NMDA open-channel antagonist improves clinical rating scores in a multiple infarct embolic stroke model in rabbits. *Brain Res* **2006**, *1088*, 141-147, doi:10.1016/j.brainres.2006.02.093.
3. Hao, J.; Mdzinarishvili, A.; Abbruscato, T.J.; Klein, J.; Geldenhuys, W.J.; Van der Schyf, C.J.; Bickel, U. Neuroprotection in mice by NGP1-01 after transient focal brain ischemia. *Brain Res* **2008**, *1196*, 113-120, doi:10.1016/j.brainres.2007.11.075.
4. Jung, K.H.; Chu, K.; Lee, S.T.; Park, H.K.; Kim, J.H.; Kang, K.M.; Kim, M.; Lee, S.K.; Roh, J.K. Augmentation of nitrite therapy in cerebral ischemia by NMDA receptor inhibition. *Biochem Biophys Res Commun* **2009**, *378*, 507-512, doi:10.1016/j.bbrc.2008.11.081.
5. Montagne, A.; Hebert, M.; Jullienne, A.; Lesept, F.; Le Behot, A.; Louessard, M.; Gauberti, M.; Orset, C.; Ali, C.; Agin, V.; et al. Memantine improves safety of thrombolysis for stroke. *Stroke* **2012**, *43*, 2774-2781, doi:10.1161/STROKEAHA.112.669374.
6. Kafi, H.; Salamzadeh, J.; Beladimoghadam, N.; Sistanizad, M.; Kouchek, M. Study of the neuroprotective effects of memantine in patients with mild to moderate ischemic stroke. *Iran J Pharm Res* **2014**, *13*, 591-598.
7. Kilic, U.; Yilmaz, B.; Reiter, R.J.; Yuksel, A.; Kilic, E. Effects of memantine and melatonin on signal transduction pathways vascular leakage and brain injury after focal cerebral ischemia in mice. *Neuroscience* **2013**, *237*, 268-276, doi:10.1016/j.neuroscience.2013.01.059.
8. Trotman, M.; Vermehren, P.; Gibson, C.L.; Fern, R. The dichotomy of memantine treatment for ischemic stroke: dose-dependent protective and detrimental effects. *J Cereb Blood Flow Metab* **2015**, *35*, 230-239, doi:10.1038/jcbfm.2014.188.

9. Chen, Z.Z.; Yang, D.D.; Zhao, Z.; Yan, H.; Ji, J.; Sun, X.L. Memantine mediates neuroprotection via regulating neurovascular unit in a mouse model of focal cerebral ischemia. *Life Sci* **2016**, *150*, 8-14, doi:10.1016/j.lfs.2016.02.081.
10. Chen, B.; Wang, G.; Li, W.; Liu, W.; Lin, R.; Tao, J.; Jiang, M.; Chen, L.; Wang, Y. Memantine attenuates cell apoptosis by suppressing the calpain-caspase-3 pathway in an experimental model of ischemic stroke. *Exp Cell Res* **2017**, *351*, 163-172, doi:10.1016/j.yexcr.2016.12.028.
11. Beladi Moghadam, N.; Pourheidar, E.; Ahmadpour, F.; Kafi, H.; Salamzadeh, J.; Nasiri, S.; Sistanizad, M. The effects of memantine on the serum concentrations of matrix metalloproteinases and neurologic function of patients with ischemic stroke. *J Clin Neurosci* **2021**, *90*, 268-272, doi:10.1016/j.jocn.2021.06.005.
12. Stanton, J.A.; Williams, E.I.; Betterton, R.D.; Davis, T.P.; Ronaldson, P.T. Targeting organic cation transporters at the blood-brain barrier to treat ischemic stroke in rats. *Exp Neurol* **2022**, *357*, 114181, doi:10.1016/j.expneurol.2022.114181.
13. Xiong, Z.; Chang, L.; Qu, Y.; Pu, Y.; Wang, S.; Fujita, Y.; Ishima, T.; Chen, J.; Hashimoto, K. Neuronal brain injury after cerebral ischemic stroke is ameliorated after subsequent administration of (R)-ketamine, but not (S)-ketamine. *Pharmacol Biochem Behav* **2020**, *191*, 172904, doi:10.1016/j.pbb.2020.172904.
14. Heil, L.B.B.; Braga, C.L.; Magalhaes, R.F.; Antunes, M.A.; Cruz, F.F.; Samary, C.S.; Battaglini, D.; Robba, C.; Pelosi, P.; Silva, P.L.; et al. Dexmedetomidine compared to low-dose ketamine better protected not only the brain but also the lungs in acute ischemic stroke. *Int Immunopharmacol* **2023**, *124*, 111004, doi:10.1016/j.intimp.2023.111004.
15. Cuartero, M.I.; de la Parra, J.; Perez-Ruiz, A.; Bravo-Ferrer, I.; Duran-Laforet, V.; Garcia-Culebras, A.; Garcia-Segura, J.M.; Dhaliwal, J.; Frankland, P.W.; Lizasoain, I.; et al. Abolition of aberrant neurogenesis ameliorates cognitive impairment after stroke in mice. *J Clin Invest* **2019**, *129*, 1536-1550, doi:10.1172/JCI120412.
16. Gu, S.X.; Sonkar, V.K.; Katare, P.B.; Kumar, R.; Kruger, W.D.; Arning, E.; Bottiglieri, T.; Lentz, S.R.; Dayal, S. Memantine Protects From Exacerbation of Ischemic Stroke and Blood Brain Barrier Disruption in Mild But Not Severe Hyperhomocysteinemia. *J Am Heart Assoc* **2020**, *9*, e013368, doi:10.1161/JAHA.119.013368.
17. Liang, Y.B.; Guo, Y.Q.; Song, P.P.; Zhu, Y.H.; Zhu, P.Z.; Liu, R.R.; Xu, J.M.; Zhang, Y.S. Memantine ameliorates tau protein deposition and secondary damage in the ipsilateral thalamus and sensory decline following focal cortical infarction in rats. *Neurosci Lett* **2020**, *731*, 135091, doi:10.1016/j.neulet.2020.135091.
18. Berthier, M.L.; Green, C.; Lara, J.P.; Higueras, C.; Barbancho, M.A.; Dávila, G.; Pulvermüller, F. Memantine and constraint-induced aphasia therapy in chronic poststroke aphasia. *Ann Neurol* **2009**, *65*, 577-585, doi:10.1002/ana.21597.
19. Yu, S.P.; Jiang, M.Q.; Shim, S.S.; Pourkhodadad, S.; Wei, L. Extrasynaptic NMDA receptors in acute and chronic excitotoxicity: implications for preventive treatments of ischemic stroke and late-onset Alzheimer's disease. *Mol Neurodegener* **2023**, *18*, 43, doi:10.1186/s13024-023-00636-1.
20. Yu, S.P.; Gu, X.; Jiang, M.Q.; Sastry, A.; Wu, L.; Li, Y.; Wei, L. Combined Preventive and Preconditioning Treatments for the Comorbidity of Alzheimer's Disease and Ischemic Stroke in a GluN3A Knockout Mouse and a 5xFAD Mouse. *Cells* **2025**, *14*, doi:10.3390/cells14231871.

21. Zhang, X.; Tian, H.; Bo, H.; Zhong, L. NMDAR inhibitor preconditioned mesenchymal stromal cell-derived extracellular vesicles enhance post-stroke recovery by targeting excitotoxicity and neuronal regeneration. *Front Cell Neurosci* **2025**, *19*, 1608615, doi:10.3389/fncel.2025.1608615.
22. Abdoulaye, I.A.; Wu, S.S.; Chibaatar, E.; Yu, D.F.; Le, K.; Cao, X.J.; Guo, Y.J. Ketamine Induces Lasting Antidepressant Effects by Modulating the NMDAR/CaMKII-Mediated Synaptic Plasticity of the Hippocampal Dentate Gyrus in Depressive Stroke Model. *Neural Plast* **2021**, *2021*, 6635084, doi:10.1155/2021/6635084.
23. Zhang, L.M.; Wu, Z.Y.; Liu, J.Z.; Li, Y.; Lv, J.M.; Wang, L.Y.; Shan, Y.D.; Song, R.X.; Miao, H.T.; Zhang, W.; et al. Subanesthetic dose of S-ketamine improved cognitive dysfunction via the inhibition of hippocampal astrocytosis in a mouse model of post-stroke chronic stress. *J Psychiatr Res* **2023**, *158*, 1-14, doi:10.1016/j.jpsychires.2022.12.010.
24. Tian, J.; Xie, Y.; Ye, S.; Hu, Y.; Feng, J.; Li, Y.; Lou, Z.; Ruan, L.; Wang, Z. S-ketamine ameliorates post-stroke depression in mice via attenuation of neuroinflammation, synaptic restoration, and BDNF pathway activation. *Biochem Biophys Res Commun* **2025**, *769*, 151965, doi:10.1016/j.bbrc.2025.151965.
25. Milani, D.; Knuckey, N.W.; Anderton, R.S.; Cross, J.L.; Meloni, B.P. The R18 Polyarginine Peptide Is More Effective Than the TAT-NR2B9c (NA-1) Peptide When Administered 60 Minutes after Permanent Middle Cerebral Artery Occlusion in the Rat. *Stroke Res Treat* **2016**, *2016*, 2372710, doi:10.1155/2016/2372710.
26. Milani, D.; Cross, J.L.; Anderton, R.S.; Blacker, D.J.; Knuckey, N.W.; Meloni, B.P. Delayed 2-h post-stroke administration of R18 and NA-1 (TAT-NR2B9c) peptides after permanent and/or transient middle cerebral artery occlusion in the rat. *Brain Res Bull* **2017**, *135*, 62-68, doi:10.1016/j.brainresbull.2017.09.012.
27. Milani, D.; Cross, J.L.; Anderton, R.S.; Blacker, D.J.; Knuckey, N.W.; Meloni, B.P. Neuroprotective efficacy of poly-arginine R18 and NA-1 (TAT-NR2B9c) peptides following transient middle cerebral artery occlusion in the rat. *Neurosci Res* **2017**, *114*, 9-15, doi:10.1016/j.neures.2016.09.002.
28. Xu, Y.; Xu, L.; Xu, C.; Zhao, M.; Xu, T.; Xia, L.; Wu, Y.; Cao, Y.; Han, Z. PSD-95 inhibitor Tat-NR2B9c (NA-1) protects the integrity of the blood-brain barrier after transient middle artery occlusion in rats by downregulating matrix metalloprotease-9 and upregulating endothelial nitric oxide synthase. *Brain Res Bull* **2024**, *206*, 110836, doi:10.1016/j.brainresbull.2023.110836.
29. Yang, X.; Zhao, J.; Tian, H.; Nie, X.; Zheng, L.; Liu, X.; Wei, Z.Z.; Ding, Y.; Liu, L. Impact of NA-1 on Pericyte-Driven Vasoconstriction and Its Role in No-Reflow During Cerebral Ischemia-Reperfusion. *CNS Neurosci Ther* **2025**, *31*, e70409, doi:10.1111/cns.70409.
30. Cook, D.J.; Teves, L.; Tymianski, M. A translational paradigm for the preclinical evaluation of the stroke neuroprotectant Tat-NR2B9c in gyrencephalic nonhuman primates. *Sci Transl Med* **2012**, *4*, 154ra133, doi:10.1126/scitranslmed.3003824.
31. Meloni, B.P.; South, S.M.; Gill, D.A.; Marriott, A.L.; Deziel, R.A.; Jacques, A.; Blacker, D.J.; Knuckey, N.W. Poly-Arginine Peptides R18 and R18D Improve Functional Outcomes After Endothelin-1-Induced Stroke in the Sprague Dawley Rat. *J Neuropathol Exp Neurol* **2019**, *78*, 426-435, doi:10.1093/jnen/nlz014.

32. Meythaler, J.M.; Brunner, R.C.; Johnson, A.; Novack, T.A. Amantadine to improve neurorecovery in traumatic brain injury-associated diffuse axonal injury: a pilot double-blind randomized trial. *J Head Trauma Rehabil* **2002**, *17*, 300-313, doi:10.1097/00001199-200208000-00004.
33. Barra, M.E.; Izzy, S.; Sarro-Schwartz, A.; Hirschberg, R.E.; Mazwi, N.; Edlow, B.L. Stimulant Therapy in Acute Traumatic Brain Injury: Prescribing Patterns and Adverse Event Rates at 2 Level 1 Trauma Centers. *J Intensive Care Med* **2020**, *35*, 1196-1202, doi:10.1177/0885066619841603.
34. Tracy, B.M.; Silverman, M.E.; Cordero-Caballero, C.; Durr, E.A.; Gelbard, R.B. Dual Neurostimulant Therapy May Optimize Acute Neurorecovery for Severe Traumatic Brain Injuries. *J Surg Res* **2021**, *268*, 546-551, doi:10.1016/j.jss.2021.07.037.
35. Badre, D.; Elbeialy, M.A.K.; Fathy, M. Citicoline-Amantadine Trial in Traumatic Brain Injury: A Prospective Randomized Study. *J Neurotrauma* **2026**, *43*, 68-77, doi:10.1177/08977151251375914.
36. Schmitt, B.; Bauersfeld, U.; Fanconi, S.; Wohlrab, G.; Huisman, T.A.; Bandtlow, C.; Baumann, P.; Superti-Furga, A.; Martin, E.; Arbenz, U.; et al. The effect of the N-methyl-D-aspartate receptor antagonist dextromethorphan on perioperative brain injury in children undergoing cardiac surgery with cardiopulmonary bypass: results of a pilot study. *Neuropediatrics* **1997**, *28*, 191-197, doi:10.1055/s-2007-973699.
37. Comi, A.M.; Highet, B.H.; Mehta, P.; Hana Chong, T.; Johnston, M.V.; Wilson, M.A. Dextromethorphan protects male but not female mice with brain ischemia. *Neuroreport* **2006**, *17*, 1319-1322, doi:10.1097/01.wnr.0000220136.98918.41.
38. Shear, D.A.; Williams, A.J.; Sharrow, K.; Lu, X.C.; Tortella, F.C. Neuroprotective profile of dextromethorphan in an experimental model of penetrating ballistic-like brain injury. *Pharmacol Biochem Behav* **2009**, *94*, 56-62, doi:10.1016/j.pbb.2009.07.006.
39. Posod, A.; Pinzer, K.; Urbanek, M.; Wegleiter, K.; Keller, M.; Kiechl-Kohlendorfer, U.; Griesmaier, E. The common antitussive agent dextromethorphan protects against hyperoxia-induced cell death in established in vivo and in vitro models of neonatal brain injury. *Neuroscience* **2014**, *274*, 260-272, doi:10.1016/j.neuroscience.2014.05.059.
